# Heterotypic interfacial tension between oncogenic and wild-type populations forms the mechanical basis of tissue-specific oncogenesis in epithelia

**DOI:** 10.7554/eLife.106893

**Published:** 2026-08-06

**Authors:** Amrapali Datta, Phanindra Dewan, Aswin Anto Puthoor, Tanya Chhabra, Tanishq Tejaswi, Sindhu Muthukrishnan, Akshar Rao, Sumantra Sarkar, Medhavi Vishwakarma

**Affiliations:** 1 Department of Bioengineering, Indian Institute of Science Bengaluru India; 2 Department of Physics, Indian Institute of Science Bengaluru India; 3 Department of Physics, Syracuse University Syracuse United States; Institut Curie, CNRS UMR168 France; CNRS France

**Keywords:** mammalian cells, lung epithelial cells, breast epithelial cells, Other

## Abstract

Why does the same oncogenic mutation drive tumor formation in some tissues but not in others? While cancer driver mutations are well documented, their tissue-specific effects remain largely attributed to genetic factors, leaving the biophysical aspects underexplored. Here, we demonstrate that mechanical interactions between newly transformed and wild-type cells are critical in determining survival and growth of HRas^V12^ mutants in human mammary and bronchial epithelia, producing contrasting outcomes in the two tissues. In mammary epithelium, isolated mutants are extruded – typical of epithelial defense against cancer – while mutant groups become spatially confined in kinetically arrested, jammed clusters, marked by an actomyosin belt at the interface. In contrast, bronchial epithelium permits persistent spreading of the mutants, which form long protrusions regardless of colony size. Furthermore, oncogenic clusters in the two tissues exhibit distinct biophysical properties, including variations in cell shapes, intracellular pressure, cell-cell tension, and cellular motility. Using a cell shape-tension coupled bi-disperse vertex model, we reveal that interfacial tension at mutant-wild-type boundaries dictates whether mutants are eliminated, restrained, or expanded. Additionally, modulating the heterotypic interfacial tension alters mutant cluster fates. Together, our findings uncover a mechanical basis for tissue-specific oncogenesis by highlighting how interfacial mechanics between mutants and wild-type populations regulate tumor initiation and progression.

## Introduction

Cancer has traditionally been understood to be a consequence of deregulated cell proliferation driven by genetic alterations that enable clonal expansion and tumor formation (Hanahan and Weinberg, 2000). Interestingly, genomic analysis reveals that many tumor suppressor genes and oncogenes exhibit tissue specificity, meaning they are altered in some cancers but not others (Schneider et al., 2017; Haigis et al., 2019; Bianchi, 2020). Current research suggests that a combination of intrinsic biological factors (Bianchi, 2020), such as differential epigenetic changes (Haigis et al., 2019; Yamashita, 2018) and aneuploidy patterns (Beroukhim et al., 2010; Zack et al., 2013; Davoli et al., 2013), as well as extrinsic factors, such as the tumor microenvironment, including the presence of tissue-specific immune cells (Salmon et al., 2019; Lehmann et al., 2017), influences tissue-specific cancer outcomes. However, a relatively underexplored yet crucial aspect of tissue-specific oncogenesis is the mechanical interaction between normal and cancerous cells. To this end, recent studies have highlighted a tumor-suppressive behavior in epithelial tissues known as epithelial defense against cancer (EDAC) (Kajita and Fujita, 2015), where transformed cells are expelled from the tissue by surrounding normal cells (Ohoka, 2015; Kajita et al., 2014; Hogan et al., 2009). However, EDAC can fail (Hogan et al., 2009), allowing mutant cells to evade extrusion when the microenvironment becomes tumor-permissive, such as in conditions of increased stiffness (Pothapragada, 2022) or inflammation due to a high-fat diet (Sasaki, 2018). The success or failure of EDAC is closely linked to changes in cellular contractility (Ohoka, 2015; Kajita et al., 2014; Hogan et al., 2009) and cell-cell adhesions (Leung and Brugge, 2012), suggesting that the underlying biophysical properties of the tissue play a decisive role in determining the outcome of cell competition. Since these properties can vary across different epithelial tissues, tissue-specific differences in cancer susceptibility may be explained by the varying ability of epithelia to manage newly transformed cells. However, despite these advances, the mechanical basis of tissue-specific oncogenesis remains missing. Specifically, how differences in cellular mechanics at the interface between oncogenic and wild-type populations influence tumor initiation and progression remains poorly understood. Addressing this gap could not only enhance our understanding of cancer biology but also open new avenues for tissue-specific therapeutic interventions by identifying what makes certain tissues more permissive to tumor formation.

To this end, we investigated and compared the biophysical factors influencing early tumorigenesis in human mammary and bronchial epithelial monolayers by sporadically transfecting a subset of healthy cells into oncogenic cells expressing a constitutively active HRas^V12^ protein. We then tracked the fate of these transfected cells and their interactions with the wild-type counterparts in real time via live imaging. Strikingly, HRas^V12^-mutant cells exhibited contrasting behaviors in the two tissues. In mammary epithelium, isolated transformed cells were frequently extruded. However, when present in groups, extrusion of oncogenic cells was not seen, but, instead, they remained spatially confined in a jammed state. In contrast, in bronchial epithelium, single as well as groups of HRas^V12^-expressing cells persisted and exhibited unrestricted expansion over time, regardless of cluster size. To explain these findings, we employed a bi-disperse vertex model of epithelia (Alt et al., 2017; Barton et al., 2017; Fletcher, 2014; Fletcher et al., 2013) and demonstrated that differences in interfacial tension between wild-type and transformed populations play a crucial role in determining whether mutant cells are eliminated, restrained, or are allowed to persist and expand. Taken together, these findings provide novel insights into the differential outcomes of competitive cellular interactions in the precancer stages and uncover a mechanical basis for tissue-specific oncogenesis.

## Results

### Singlets and groups of HRas^V12^ oncogenic mutants show tissue-specific fates in epithelia

**Figure 1.**
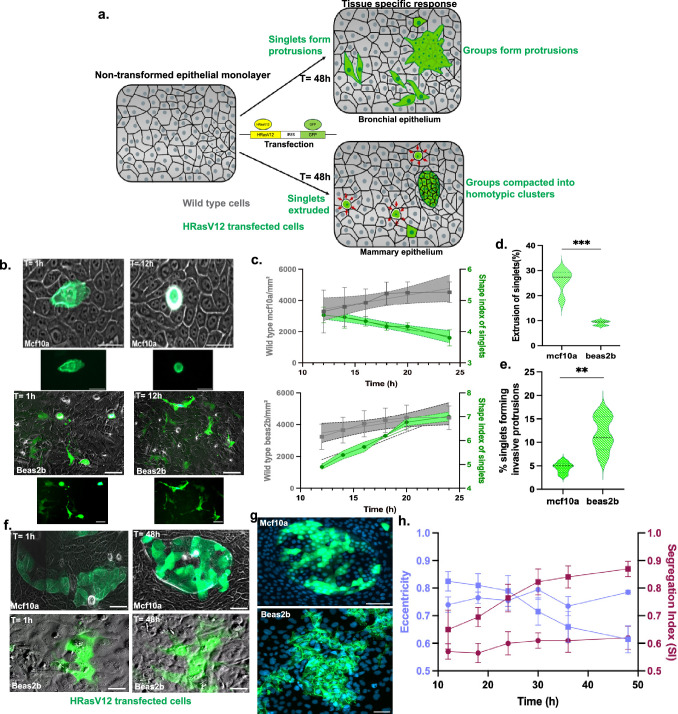
Tissue-specific outcomes of HRas^V12^ oncogenic mutants in epithelial monolayers**.** (**a**) Schematic representation of the experimental setup and outcome. (**b**) Representative images showing extrusion of single oncogenic cells (singlets) in mammary epithelium (*upper panel*), but protrusive behavior of the same oncogenic singlets in bronchial epithelium (*bottom panel*). (**c**) Quantification of shape indices of HRas^V12^ singlets showing a reduction in shape indices, with increasing wild-type cell density in mammary epithelium (*upper panel*), but the opposite trend in bronchial epithelium (*bottom panel*), where HRas^V12^ cells continue to spread. Shaded regions indicate the results of simple linear regression analysis with 95% confidence intervals. (**d**) Extrusion rates of HRas^V12^ singlets are significantly higher in mammary epithelium, while (**e**) the percentage of HRas^V12^ cells forming protrusions is markedly higher in bronchial epithelium. Representative data are plotted from one of three independent experiments, with the median shown as a bold, dashed line and the first and third quartiles are shown as thin dashed lines. Statistical significance was calculated using an unpaired t-test with Welch’s correction. (**f**) Behavior of HRas^V12^ oncogenic clusters in the two tissues. In mammary epithelium (*upper panel*), clusters become spatially confined with a smooth, circular interface with the wild-type population, while in bronchial epithelium (*bottom panel*), HRas^V12^ clusters expand, forming long protrusions over time. (**g**) Immunofluorescence images of HRas^V12^ clusters in mammary and bronchial epithelia, highlighting differences in spreading patterns in the two epithelia. (**h**) Quantification of cluster segregation: mammary epithelium exhibits a higher segregation index and reduced eccentricity compared to bronchial epithelium, indicating greater spatial confinement of oncogenic clusters. The segregation index (SI), defined as the average ratio of homotypic and all cell neighbors, quantifies the degree of demixing and is averaged over all cells inside an oncogenic cluster. Data are mean ± sem and plotted from different clusters from one representative experiment (*scale bars = 50 μm*).

To explore the differences in the premalignant stages across epithelial tissues, we sporadically transfected monolayers of the two non-transformed human epithelial cell lines, MCF10A (mammary) and BEAS2B (bronchial), with a plasmid construct that constitutively expresses HRas^V12^ along with the GFP reporter (Figure 1a). We then monitored the cellular dynamics over 90 hr. Consistent with previous studies reporting extrusion of single HRas^V12^ mutants in MDCK monolayers (Kajita and Fujita, 2015; Ohoka, 2015; Kajita et al., 2014; Hogan et al., 2009; Pothapragada, 2022; Gupta et al., 2024), we observed that isolated HRas^V12^-transfected cells (singlets) surrounded by wild-type neighbors were extruded from the mammary epithelium (Figure 1b, upper panel, Figure 1—video 1). This process correlated with a reduction in shape indices of oncogenic mutants as the density of wild-type cells around them increased (Figure 1c, upper panel). Interestingly, this well-known mechanism of EDAC was impaired in BEAS2B cells, where HRas^V12^ mutants evaded extrusion (Figure 1b, lower panel, Figure 1d), and wild-type cell compaction did not restrict their growth (Figure 1c, lower panel, Figure 1—video 1). Instead, the mutants in the bronchial epithelium continued to proliferate and exhibited increased elongation with long protrusions (Figure 1c, lower panel, Figure 1e, Figure 1—video 1). Given that cancers frequently arise from fields of oncogenic mutants rather than isolated mutated cells (Sinjab et al., 2020; Franklin et al., 1997; Curtius et al., 2018), we also investigated whether these differential dynamics of mutant singlets would also extend to groups of oncogenic mutants, potentially enabling more aggressive HRas^V12^ growth in bronchial tissue compared to mammary tissue. By adjusting cell seeding conditions, we generated larger groups of HRas^V12^-transfected cells in both MCF10A and BEAS2B monolayers and then tracked the dynamics of these groups over time. In the mammary epithelium, wild-type cells were unable to extrude groups of HRas^V12^-transfected cells but instead confined them into compact, circular clusters with smooth, rounded interfaces (Figure 1f, upper panel, Figure 1g, upper panel, Figure 1—video 2). As a result, oncogenic clusters gradually demixed from the surrounding wild-type MCF10A population and remained spatially constrained (Figure 1h). In contrast, HRas^V12^ clusters in the bronchial epithelium gradually spread outward (Figure 1f, lower panel, Figure 1g, lower panel, Figure 1—video 2), and formed protrusive lamellipodia and developed an irregular interface with surrounding wild-type cells (Figure 1g, lower panel). Together, these striking differences in the dynamics of HRas^V12^ oncogenic cells demonstrate that activation of the same oncogene can lead to distinct outcomes across tissues in terms of growth, spreading, and extrusion. This prompted us to further investigate the unique biophysical mechanisms driving these variations.

**Figure 1—video 1.** An HRas^V12^ singlet is extruded from MCF10A monolayer but persists in BEAS2B monolayer, multiplies and forms long protrusions. Scale bar = 20 μm.

**Figure 1—video 2.** A group of HRas^V12^-transfected cells gradually gets confined into a compact cluster in MCF10A monolayer and forms a smooth interface with the wild-type population, while a group of HRas^V12^-transfected cells gradually grows in size in BEAS2B monolayer, forming a jagged interface with the wild-type population along with protrusions. Scale bar = 50 μm.

### Biophysical analysis reveals demixing via jamming of oncogenic clusters in MCF10A but unjamming and protrusive growth in BEAS2B

**Figure 2.**
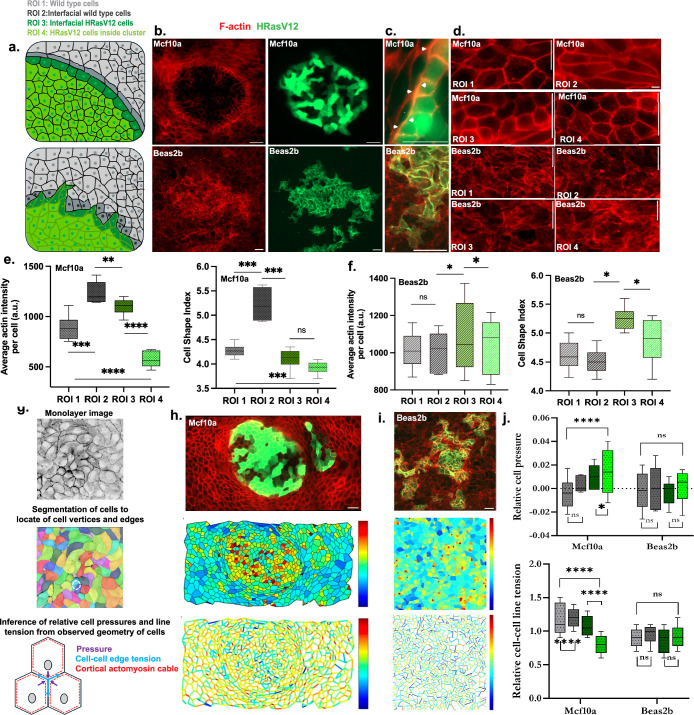
Distinct mechanical signatures of HRas^V12^ clusters in mammary and bronchial epithelia. (**a**) Representative images showing regions of interest (ROIs) selected for analysis in mammary (*upper panel*) and bronchial epithelia (*lower panel*). (**b**) Immunofluorescence images of HRas^V12^ clusters stained for F-actin in mammary (*upper panel*) and bronchial epithelia (*lower panel*). (**c**) Oncogenic cluster-wild-type interface showing distinct actin belt in mammary marked by white arrows (*upper panel*) and absence of it in bronchial epithelia (*lower panel*). (**d**) ROI-based F-actin-stained regions of wild-type and oncogenic cells in mammary (upper panel) and bronchial epithelia (lower panel), revealing distinct shape differences in different regions – in mammary epithelia, wild-type cells at the interface show elongated shapes (ROI2), and oncogenic cells inside the cluster show jammed shapes (ROI4), in comparison to wild-type cells at a random location (ROI1), while in bronchial epithelia, oncogenic cells (ROI3 and ROI4) show more elongated shapes compared to wild-type BEAS2B cells (**e**). (**f**) Quantifications of F-actin intensities and shape indices in mammary epithelia (**e**) and bronchial epithelia (**f**) across the four ROIs confirming jammed oncogenic clusters, with unjamming at the interface, in mammary epithelia and unjammed clusters in bronchial epithelia. (**g**) Schematic illustrating the Bayesian force inference pipeline used to estimate relative intracellular pressure and cell-cell edge tension. (**h**) Heatmaps depicting relative cell pressures and cell-cell edge tensions in mammary epithelium, revealing localized mechanical heterogeneity. (**i**) Heatmaps of relative cell pressures and edge tensions in bronchial epithelium, showing a distinct mechanical landscape compared to mammary epithelium. (**j**) Quantification of relative intracellular pressures and cell-cell edge tensions across the four ROIs in mammary (upper panel) and bronchial epithelia (lower panel), highlighting tissue-specific differences. All data represented as mean ± sem in box and whisker plots and are plotted from one out of three independent experiments with whiskers extending from the minimum to the maximum values, showing the full range of the dataset including outliers. Line represents the median. Statistical significance was calculated using an unpaired t-test with Welch’s correction. *Scale bars = 20 μm.*

To understand the mechanisms driving the differential fates of oncogenic fields in the two epithelial tissues, we mapped the biophysical signatures of wild-type-mutant interactions by subdividing the images into four regions of interest for both monolayers: ROI1, wild-type cells distant from oncogenic cells; ROI2, interfacial wild-type cells in direct contact with oncogenic cells; ROI3, oncogenic cells at the cluster boundaries in direct contact with wild-type cells; and ROI4, oncogenic cells in contact with other oncogenic cells (Figure 2a). We then compared the F-actin levels and shapes of both mutant and wild-type populations across these ROIs. Additionally, we employed Bayesian force inference, as previously established (Ishihara and Sugimura, 2012), to infer the differential tissue stresses associated with cluster formation from the observed cell geometries. Interestingly, the differences across these parameters were apparent not only between wild-type and oncogenic counterparts but also based on the spatial localization of the cells, with cells at the oncogenic-wild-type interface exhibiting distinct behaviors, regardless of their origin.

**Figure 2—figure supplement 1.**
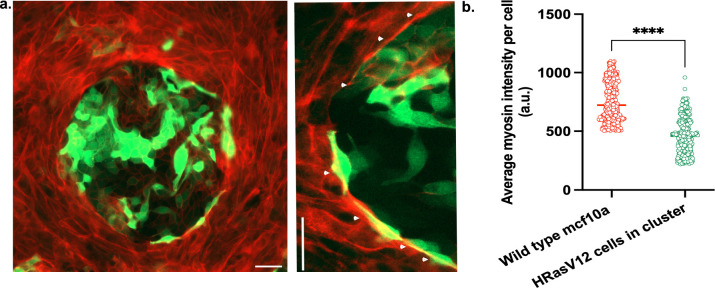
Myosin belt and per-cell myosin intensities. (**a**) Images of HRas^V12^ clusters in mammary epithelia stained for phosphomyosin (left panel) and phosphomyosin belt around the cluster marked with white arrowheads (right panel). (**b**) Plot showing lower phosphomyosin levels inside oncogenic clusters in MCF10A. Representative data is plotted from one experiment with the median shown as a bold line. Statistical significance was calculated using an unpaired t-test with Welch’s correction. *Scale bars = 50 μm*.

**Figure 2—figure supplement 2.**
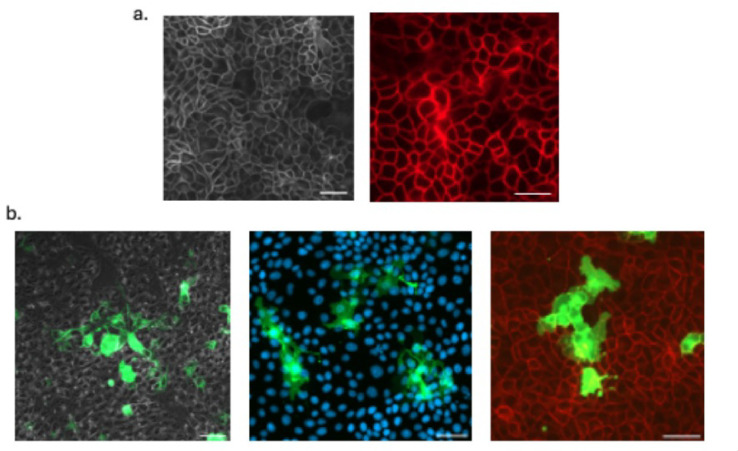
HRas^V12^ clusters show invasive phenotype in MDCK monolayer. (**a**) Representative images of wild-type MDCK monolayers stained for F-actin (left) and E-cadherin (right), showing intact cell-cell junctions. (**b**) HRas^V12^ clusters expand, forming long protrusions over time. *Scale bars = 50 μm*.

**Figure 2—figure supplement 3.**
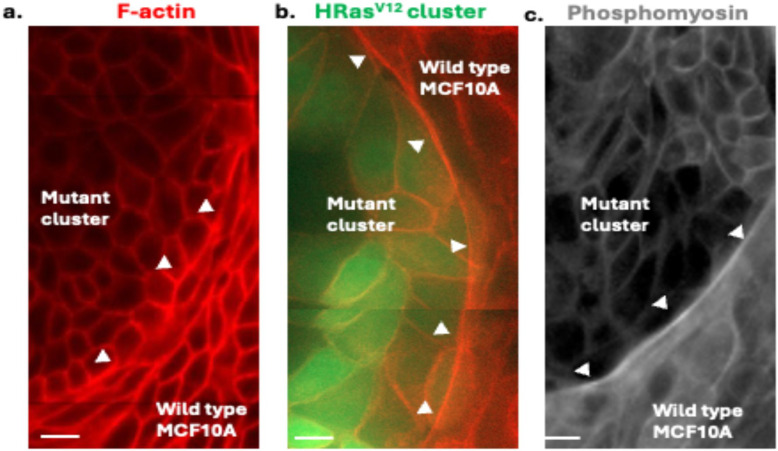
Actomyosin enrichment at the interface of HRas^V12^ clusters and wild-type MCF10A. (**a**) Images of HRas^V12^ clusters in mammary epithelia stained for F-actin, showing higher F-actin deposition at heterotypic interfaces. (**b**) Representative images of HRas^V12^ clusters in mammary epithelia stained for phosphomyosin, showing higher myosin deposition at the heterotypic interface. *Scale bars = 10 μm*.

In mammary epithelium, oncogenic clusters were surrounded by a contractile actomyosin belt (Figure 2b, upper panel, Figure 2c, upper panel), shown by a prominent deposition of F-actin and phosphomyosin at heterotypic (oncogenic cluster-wild-type population) interfaces (Figure 2—figure supplement 1) coupled with drastically low F-actin levels inside the clusters (Figure 2b, upper panel, Figure 2e, left panel), suggesting that the de-mixing of oncogenic cells in mammary tissue is likely driven by changes in cytoskeletal mechanics. Additionally, HRas^V12^ mutant clusters adopted an invasive, protrusive morphology in MDCK epithelial monolayer (Figure 2—figure supplement 2). Further, oncogenic clusters in mammary epithelium (ROI4) also exhibited low cell shape indices (Figure 2d, upper panel, Figure 2e, right panel), high internal pressure, and low line tension (Figure 2h and j) – characteristics indicative of jamming (Atia et al., 2018). In epithelial tissues, the jamming-unjamming transition is closely linked to changes in cell shape (Atia et al., 2018) and is often quantified using the cell shape index (the ratio of cell perimeter to the square root of area), where lower shape indices correspond to jammed, solid-like states, and higher shape indices reflect unjammed, fluid-like tissue behavior. Notably, both oncogenic cells and wild-type MCF10A cells at the cluster interface (ROI2 and ROI3) showed F-actin enrichment, while the interfacial wild-type cells showed elongated shapes (Figure 2d, upper panel, Figure 2e), indicating that interfacial cellular mechanics play a key role in the demixing process as the wild-type cells encircle the oncogenic mutants. This interfacial region enriched with F-actin also contained phosphomyosin (Figure 2—figure supplement 3), indicating that a contractile actomyosin belt drives the compaction of oncogenic clusters in mammary epithelia. In contrast, bronchial epithelium exhibited no actin belt formation at the wild-type-oncogenic cell interface, and no traits of jamming were observed in the oncogenic clusters (Figure 2b, lower panel, Figure 2c, lower panel). Instead, oncogenic cells continued to proliferate, displaying elongation measured via higher shape indices and high F-actin expression (Figure 2d, lower panel, Figure 2f) – indicative of unjamming behavior (Atia et al., 2018; Park et al., 2015). The relative internal cell pressure and cell-cell line tension inferred with Bayesian force inference showed slight or no modulation in the oncogenic clusters, given the general elongated shapes of wild-type cells in BEAS2B cells (Figure 2i and j). Together, these findings highlight distinct interfacial mechanics between oncogenic cells and their wild-type neighbors in both MCF10A and BEAS2B monolayers, resulting in differing outcomes in mutant colony growth.

### PIV analysis reveals kinetic arrest of oncogenic clusters in MCF10A coupled with tangential motion of interfacial cells

**Figure 3.**
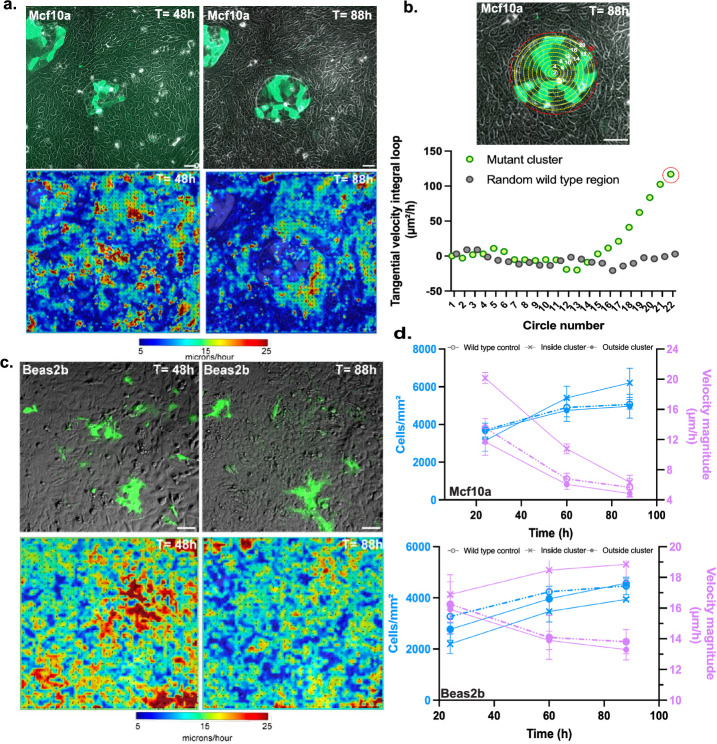
Particle image velocimetry (PIV) reveals distinct cellular movement patterns in HRas^V12^ clusters in the two monolayers. (**a**), (**b**) Representative snapshots of HRas^V12^ clusters, and the corresponding PIV maps, in mammary (**a**) and bronchial epithelium (**b**), as the density of wild-type cells increases, showing wild-type cells along the cluster-wild-type interface exhibit a higher velocity attributed to tangential motion in mammary epithelium (**a**), but undergo a more kinetically arrested state in bronchial epithelium (**b**). (**c**) Oncogenic cluster in MCF10A monolayer with concentric circles drawn from the center of the cluster, radially outward till its boundary (upper panel) with the red (highlighted) circle representing the one along which tangential velocity was the highest and plot showing tangential motion (expressed as a function of the circles), revealing highest tangential motion (data point highlighted with red) along the interface of oncogenic cluster with the wild-type cells in mammary epithelia (lower panel). No significant tangential motion in random wild-type region. (**c**) Representative snapshots of HRas^V12^ clusters in bronchial epithelium at the same two time points and the corresponding PIV velocity maps. (**d**) Quantification of cellular velocities in local regions as cell densities reach confluency showing oncogenic cells clustered in mammary epithelium (*top panel*) jam and show a significant reduction in velocities. In bronchial epithelium (*bottom panel*), while wild-type cells show reduced movement as cell density increased, transfected cells continued to show higher motion, signifying that jamming of wild-type cells has no effect on mutants. All data are represented as mean ± sem plotted from different clusters from one representative experiment. *Scale bars = 50 μm*.

To further probe the cellular dynamics that lead to the contrasting fates of the oncogenic mutant colonies in the two tissues, we analyzed cellular velocities during the demixing of oncogenic cells, using particle image velocimetry (PIV) on consecutive image pairs from mammary and bronchial monolayers. Interestingly, velocity maps in samples imaged for mutant cluster-wild-type dynamics in MCF10A (Figure 3—video 1) revealed an unusual movement pattern at confluency: wild-type MCF10A cells exhibited tangential motion around oncogenic clusters (Figure 3c), which coupled with a drastic reduction in the velocity of oncogenic cells (Figure 3a, lower panel, Figure 3d), likely caused their compaction and jamming into clusters. Notably, this tangential movement was unique to wild-type MCF10A cells surrounding oncogenic clusters and was absent in other regions across the monolayer (Figure 3c), as well as under control conditions, i.e., in the absence of oncogenic mutants (Figure 3—figure supplement 1, Figure 3—video 2), in both of which a typical velocity drop was observed in MCF10A cells due to density-induced jamming (Atia et al., 2018; Park et al., 2015; Sadati et al., 2013; Mitchel et al., 2020; Figure 3d, upper panel, Figure 3—figure supplement 1a). These results aligned with the observed differences in cell shape indices, where oncogenic clusters underwent jamming, while wild-type cells at the interface exhibited unjamming behavior (Sadati et al., 2013; Mitchel et al., 2020). In contrast, in the bronchial epithelium, mutant clusters remained unjammed, even as surrounding wild-type cells became more crowded (Figure 3b and d, lower panel, Figure 3—video 3). Additionally, no tangential cellular movement was detected around the oncogenic clusters in the bronchial epithelium. These velocity maps showed negligible deviations from wild-type (control) BEAS2B velocity maps (Figure 3—video 4). Together, these findings suggest that mutant clusters carrying the same oncogene can exhibit drastically different behaviors depending on the epithelial tissue type. Specifically, differences in the dynamics of the interfacial cells drive the demixing and jamming of HRas^V12^ oncogenic cells in mammary epithelium while promoting protrusive, unjammed growth in bronchial epithelium.

**Figure 3—figure supplement 1.**
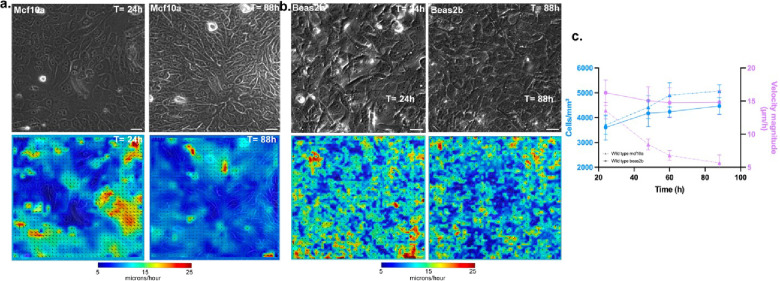
Particle image velocimetry (PIV) maps of wild-type mammary and bronchial epithelial monolayers with increasing cell density. (**a**) Representative time-lapse images of wild-type (control) mammary epithelium at two different time points, with corresponding PIV velocity maps showing a reduction in overall velocity as cell density increases. (**b**) Representative time-lapse images of wild-type (control) bronchial epithelium at two different time points, with corresponding PIV velocity maps demonstrating a similar reduction in velocity with increasing cell density. (**c**) Quantification of velocity reduction in wild-type mammary and bronchial epithelia as a function of cell density, highlighting tissue-specific differences in collective cell dynamics. All data are represented as mean ± sem plotted from different clusters from one representative experiment. *Scale bars = 50 μm*.

**Figure 3—video 1.** Particle image velocimetry (PIV) video during formation of a representative HRas^V12^ cluster in MCF10A monolayer. *Scale bar = 50 μm*.

**Figure 3—video 2.** Particle image velocimetry (PIV) video showing the dynamics of a representative wild-type MCF10A (control) monolayer without any oncogenic mutants. *Scale bar = 50 μm*.

**Figure 3—video 3.** Particle image velocimetry (PIV) video showing the dynamics of a representative HRas^V12^ cluster in BEAS2B monolayer. *Scale bar = 50 μm*.

**Figure 3—video 4.** Particle image velocimetry (PIV) video showing the dynamics of a representative wild-type BEAS2B (control) monolayer without any oncogenic mutants. *Scale bar = 50 μm*.

### Bi-disperse vertex model reveals interfacial tension between mutant and wild-type population governs tissue-specific response toward oncogenic mutants

**Figure 4.**
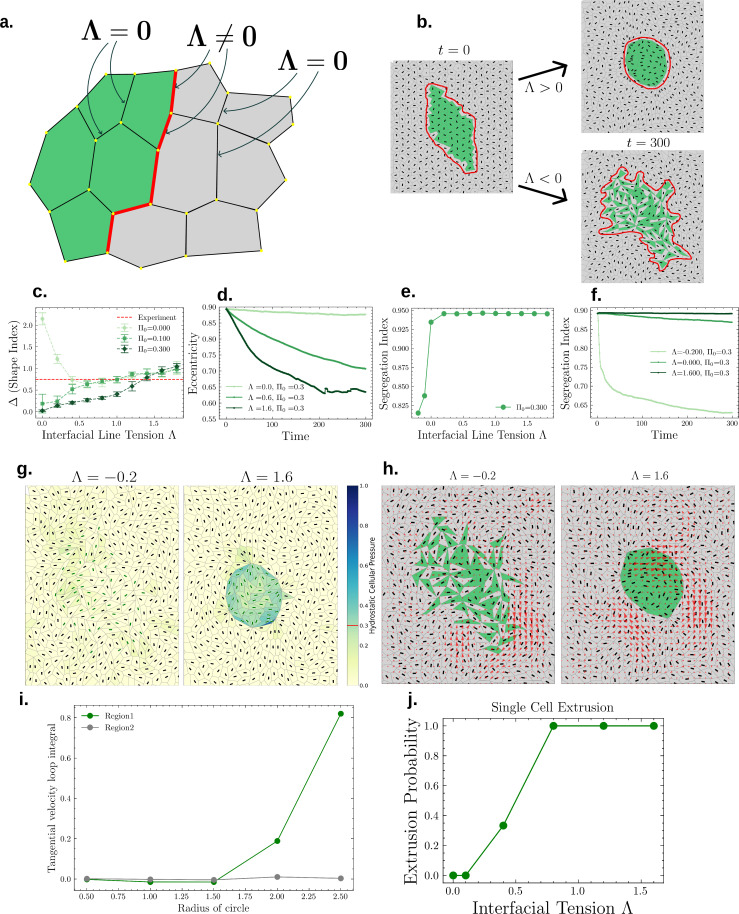
Bi-disperse vertex model reveals interfacial tension-driven segregation of oncogenic clusters. (**a**) Schematic of the bi-disperse vertex model, where interfacial line tension (red) is applied at the boundary between mutant (green) and wild-type (gray). (**b**) The value of Λ determines whether the mutant cluster remains compact or spreads into the wild-type population, where the red line gives the outline of the morphology of the mutant cluster interface. (**c**) Difference in shape index between interfacial and bulk cells as a function of Λ (interfacial tension), shown for different stress thresholds. Experimental average (red) overlaid for comparison is the difference between the shape indices for ROI2 (interface), and ROI1, ROI3, and ROI4 (away from interface) from Figure 2e. (**d**) Eccentricity of mutant clusters (given by the red lines in (**b**)) over time for different values of Λ (with Π0=0.3), indicating changes in cluster morphology. (**e**) Segregation index at *t*=300 as a function of Λ for Π0=0, quantifying the extent of cluster segregation. (**f**) Time evolution of the segregation index for different values of Λ (with Π0=0.3 *with Π0=0.3*), demonstrating interfacial tension-driven segregation. (**g**) Hydrostatic pressure maps of mutant and wild-type cells at *t*=300, with mutant cells labeled by green directors and wild-type cells by black directors. (**h**) Velocity field maps of epithelial monolayers for different values of Λ, revealing flow patterns around oncogenic clusters. (**i**) Tangential velocity loop integral for circles of varying radius centered at the mutant cluster (Region 1) and centered away from the cluster (Region 2), showing tissue-specific velocity variations. (**j**) Extrusion probability of a single mutant cell as a function of Λ, demonstrating how interfacial tension influences mutant cell fate.

To explain the differential dynamics and degrees of demixing of oncogenic clusters observed in the two tissues, we drew inspiration from the differential interfacial tension hypothesis (DITH) (Foty and Steinberg, 2005). This hypothesis suggests that cell sorting in tissues could occur due to the variations in interfacial tension, as seen in embryonic cell rearrangement, where cells regulate contractility, as well as adhesions to demix from their neighbors (Foty and Steinberg, 2005; Brodland, 2002; Canty et al., 2017). To test whether such a mechanism could be applied to the demixing of oncogenic clusters from wild-type cells, leading to tissue-specific outcomes, we employed the cell-based bi-disperse vertex model. Our model allowed us to simulate an epithelial monolayer and capture the dynamic interactions between wild-type and mutant cell populations. We analyzed the mechanics underlying the segregation dynamics of mutant clusters by examining the interfacial line tension $\Lambda _{ij}^{M\mathrm{/}WT}$ between the oncogenic and wild-type cells (Figure 4a). In our model, $\Lambda _{ij}=0$, for homotypic interfaces, i.e., wild-type-wild-type or mutant-mutant interactions, and $\Lambda _{ij}=\Lambda \neq 0$, for heterotypic interfaces between oncogenic and wild-type cells (Appendix 1—figure 1). When Λ>0, our model ensures that the interface between oncogenic and healthy cells contracts to minimize energy, leading to a reduction in total interfacial length, and when Λ<0, our model promotes a rough interface by favoring increased interface between healthy and oncogenic cells (Figure 4—video 1). The final interface shape is determined by the balance between interfacial tension and the intrinsic perimeter and area elasticity of individual cells (Figure 4b).

**Figure 4—figure supplement 1.**
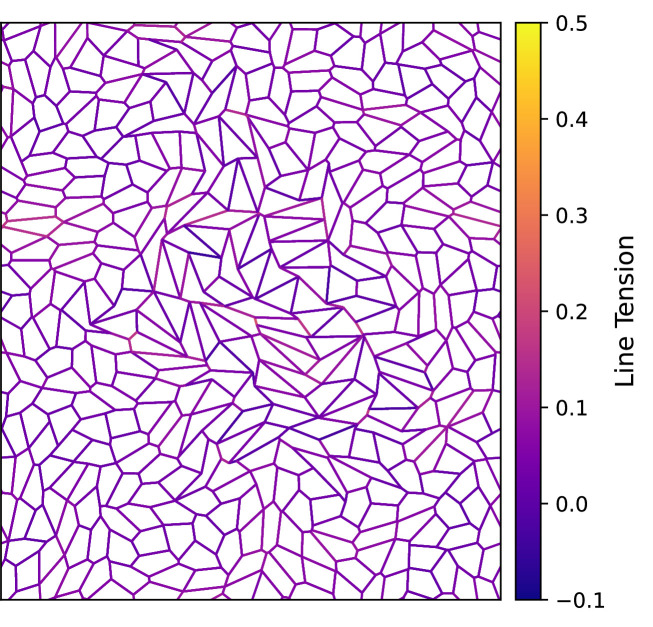
Normalized tangential velocity loop integral plotted vs. circle number (increasing radius). The integral has been normalized by the perimeter of the circle. The same trend as in the unnormalized analysis is seen. Average has been taken over eight different wild-type and mutant cluster positions.

**Figure 4—figure supplement 2.**
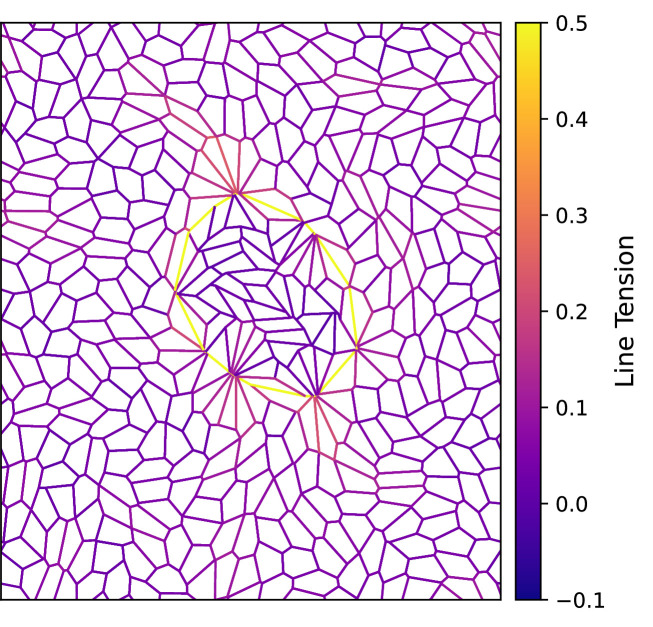
Normalized tangential velocity loop integral plotted for circle number 22 (cells at the interface of the mutant clusters) vs. time. The integral has been normalized by the perimeter of the circle. We see tangential velocity persists for over 24 hr, till the end of the time-lapse video, starting when the mutants have formed a mature, compact, rounded, well-defined cluster.

**Figure 4—figure supplement 3.**
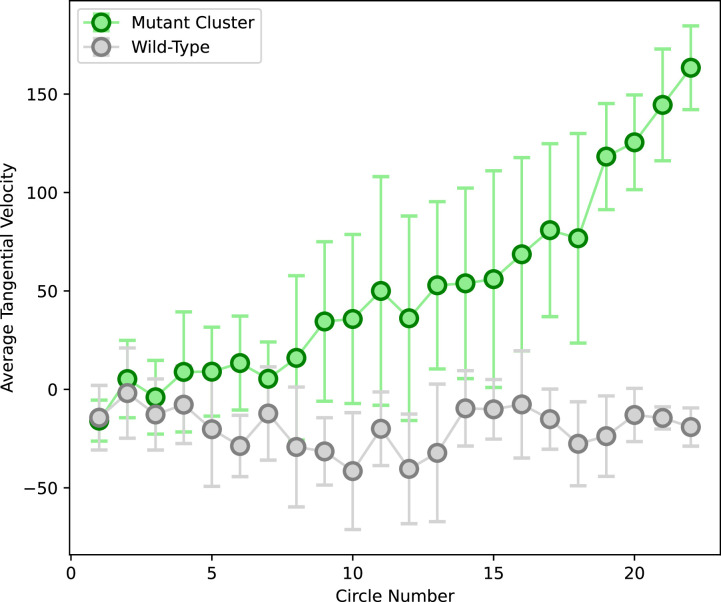
Line tensions from simulation for Λ = −0.2.

**Figure 4—figure supplement 4.**
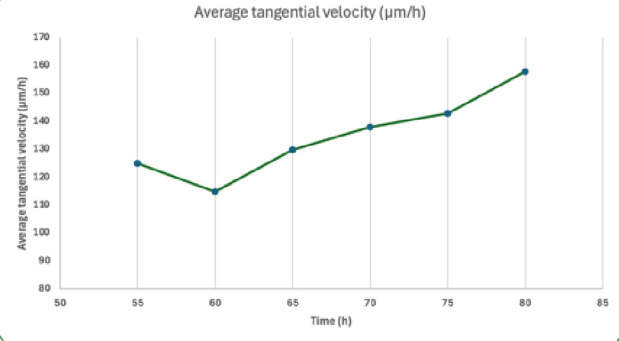
Line tensions from simulation for Λ=1.6.

**Figure 4—video 1.** Video showing output of the bi-disperse vertex model with cluster dynamics varying with interfacial tension (Λ).

To explain the observed differences in interfacial cellular dynamics, we introduced an active contribution to interfacial tension, which was coupled to the shape of cells at the interface, called shape-tension coupling (Rozman et al., 2023). This active interfacial tension is extensile, meaning it elongates cells along the interface depending on their orientation relative to the interface. It originates from the anisotropic distributions of stress fibers when the cells are under external stress. Hence, we assume that it becomes significant only when the hydrostatic pressure on the interfacial cells exceeds a threshold value $\Pi _{0}$ (Figure 4b and g).

Remarkably, our model robustly captured the experimentally observed behaviors. As a first check, we compared the experimentally observed differences in the shape indices between interfacial and non-interfacial wild-type and oncogenic cells in both mammary and bronchial epithelium with our model’s predictions. For $\Gamma =0.2,\Pi _{0}=\left \{0,0.1,0.3\right \},\Lambda \mathrm{=}\left \{0\mathrm{-}1.6\right \}$, our model accurately captured the experimental observations (Figure 4c). For stress threshold values lower than $\Pi _{0}=0.3$, we find that the differences in shape indices are non-zero even for $\Lambda \mathrm{=}0$, which is an unphysical situation. This is unphysical because we expect there to be no difference in the shape indices because, in principle, there is only one cell type for $\Lambda =0$. Thus, we have taken $\Pi _{0}=0.3$ for further analyses. To make the comparison between the model and the experiment more robust, we compared other metrics as well.

To quantify the extent of mixing between wild-type and mutant cells, we used the segregation index. Our simulations showed that when $\Lambda < 0$, the segregation index rapidly decreases over time, indicating outward protrusion of the mutant cluster. In contrast, for $\Lambda > 0$, the segregation index remained close to 1, reflecting a compact, jammed cluster (Figure 4f). Additionally, we observed that when $\Lambda > 0$, cells within the cluster experience higher internal pressure compared to when $\Lambda < 0$ (Figure 4g), consistent with experimental findings (Figure 2h and i). Line tensions along edges in the vertex model have also been calculated (Figure 4—figure supplements 1 and 2). Furthermore, flow field analysis from our simulations (Figure 4h and i) revealed that for $\Lambda > 0,$ cell movement aligns tangentially along the cluster boundary – consistent with the MCF10A behavior (Figure 4—figure supplements 3 and 4) – whereas for $\Lambda < 0$ cell motion was uniform throughout the monolayer, resembling the BEAS2B phenotype (Figure 3a and b). These comparisons further establish that different values of $\Lambda $ correspond to different cell types. The same oncogene thus might affect different cell lines differently. We also estimated the probability of single mutant cell extrusion as a function of Λ (Figure 4j), consistent with the findings from our experiments which show that isolated HRas^V12^-transfected cells are frequently extruded in mammary tissue (Figure 1b). Further, to check how actin distribution in the tissue can change in response to the interfacial tension in the model, we performed simulations of a vertex model with interfacial tension and mechanochemical feedback (Muthukrishnan et al., 2025). The mechanochemical feedback incorporates a load-dependent myosin-binding mechanism, allowing cells in the vertex model to adjust their fractions of stress fibers and cortical actin individually. The simulation snapshots matched quite well with those from experiment (Appendix 1—figure 5). Together, our findings demonstrate how interfacial tension-driven mechanics could play a crucial role in determining the competitive dynamics between wild-type and mutant populations, thus providing compelling evidence that cellular and tissue mechanics are key determinants of cell competition.

### Disrupting actomyosin belt using blebbistatin prevents demixing and compaction of mutant clusters in mammary epithelia

Since our model showed that differences in interfacial tension regulate mutant cluster dynamics, with a higher interfacial tension driving cluster compaction (Figure 4—video 1), we reasoned that this tension is mediated by the supracellular actomyosin belt (Martin, 2010) that drives the compaction and segregation of oncogenic clusters in mammary epithelium. Therefore, we investigated whether disrupting the cytoskeletal organization and impairing actomyosin belt formation would affect the demixing and compaction of oncogenic clusters. To test this, we treated MCF10A monolayers with 5 µM blebbistatin, a myosin II inhibitor, to disrupt belt formation.

**Figure 5.**
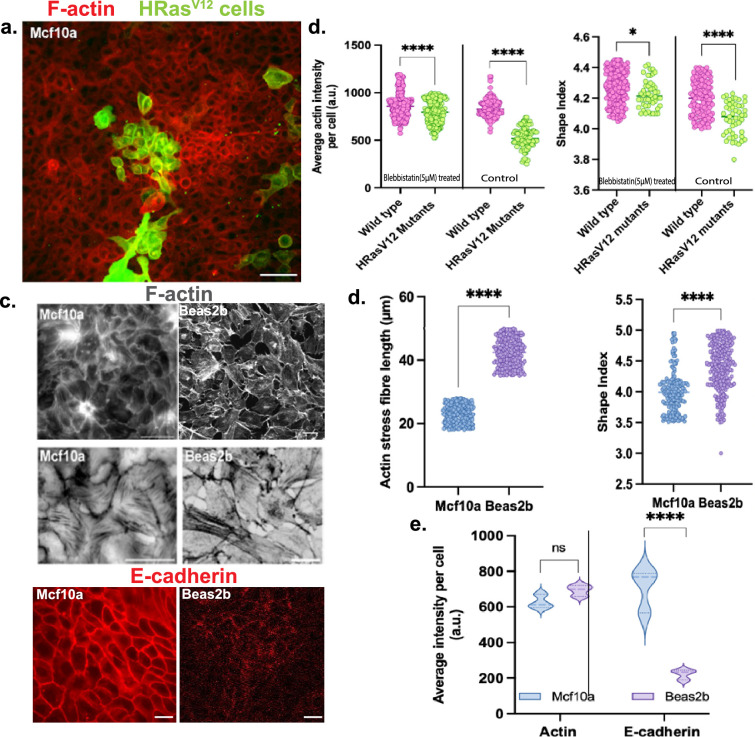
Actomyosin belt disruption prevents mutant cluster compaction in mammary epithelia. (**a**) Representative actin staining image upon blebbistatin treatment, done after oncogenic induction in mammary epithelial monolayer, showing an absence of an actin belt. (**b**) Quantification of F-actin intensities (left) and shape indices (right), showing differences between oncogenic clusters and wild-type cells in these parameters, is getting small – although not completely vanished because of low concentration of blebbistatin used for the experiment. (**c**) Representative actin and E-cadherin staining images of the wild-type monolayers showing shorter stress fibers, higher E-cadherin intensities in MCF10A wild-type monolayer, and the opposite in BEAS2B. (**d**) Plots comparing F-actin stress fiber length (left panel) and shape indices (right panel) in wild-type MCF10A and BEAS2B monolayers. Stress fiber lengths were calculated manually in ImageJ. (**e**) Plots comparing F-actin and E-cadherin levels in the two wild-type tissues. Representative data is plotted from one experiment with the median shown as a bold line. Statistical significance was calculated using an unpaired t-test with Welch’s correction. *Scale bars = 50 μm*.

As expected, blebbistatin treatment prevented mutant cluster compaction (Figure 5a), leading to a loss of their rounded morphology and restoring actin levels and shape indices of mutant cells to wild-type levels (Figure 5b). This shift resulted in mutant clusters resembling those observed in bronchial epithelia, suggesting that cytoskeletal organizations play a key role in tissue-specific differences in mutant cluster dynamics. This was also consistent with the fact that interfacial tension between cells arises from the interplay between the cortical actomyosin network and cell-cell adhesion (Manning et al., 2010).

Additionally, we looked into the inherent differences in cytoskeletal mechanics of the host tissues and observed distinct differences in actin organization between the two tissues. Wild-type bronchial and mammary epithelial cells exhibited comparable F-actin intensities (Figure 5c, upper panel, Figure 5e), although the bronchial epithelial cells had longer stress fibers (Figure 5c, middle panel, Figure 5d, left panel). These cytoskeletal differences were also coupled with significant differences in cell shapes between confluent MCF10A and BEAS2B monolayers, with wild-type BEAS2B cells exhibiting significantly higher shape indices, indicating elongated, loosely packed morphologies and a lower degree of jamming (Figure 5d, right panel). In contrast, mammary epithelial cells had lower shape indices, reflecting a more compact and tightly packed arrangement (Figure 5d, right panel). These differences were also in agreement with the PIV maps of the two wild-type tissues (Figure 3—videos 2 and 4), where the mammary monolayers underwent a sharper drop in velocities and therefore kinetic arrest, compared to the bronchial epithelia. Bronchial epithelial cells also exhibited weaker cell-cell adhesions, as indicated by reduced E-cadherin staining intensity at cell junctions compared to mammary epithelial cells (Figure 5c, bottom panel, Figure 5e). These weaker adhesions likely contributed to increased cellular motility and a diminished ability to constrain mutant clusters, in contrast to the more stable, compacted clusters observed in mammary epithelium. Together, these findings demonstrate that variations in mechanical properties, including cell shape, actin organization, and adhesion strength, govern the differential responses of mammary and bronchial epithelia to oncogenic mutants and may, in turn, influence tumor growth and progression.

## Discussion

A central challenge in cancer biology is understanding why some oncogenic mutations are more potent in certain cancers. Large-scale cancer genomics has revealed that many driver mutations are shared across epithelial cancers, yet tumor incidence, progression rates, and invasive potential vary widely between organs (Vogelstein et al., 2013; Martincorena and Campbell, 2015). These observations have traditionally been explained through tissue-specific differences in signaling pathways, differentiation states, and mutational landscapes. However, such explanations are incomplete, particularly in the context of early tumorigenesis, where oncogenic mutations are often detected in histologically normal tissues without leading to overt cancer (Vogelstein et al., 2013). This raises a fundamental question: What prevents or permits oncogenic cells from expanding at the earliest stages of cancer initiation?

The present study addresses this gap by proposing interfacial mechanics between wild-type and nascently transformed cells as a central organizing principle in early cancer initiation. Rather than focusing on oncogenic cells in isolation, our work frames tumor initiation as an emergent outcome of mutant-wild-type interactions within an epithelial collective. From this perspective, the critical determinant of oncogenic fate is not only the presence of a mutation, but how the host tissue mechanically responds to it. This conceptual shift aligns with growing recognition that epithelial tissues behave as active materials, whose collective mechanical state can either suppress or amplify local perturbations. These insights shed light on the differential susceptibility of epithelial tissues to oncogenic transformation and uncover a mechanical basis for tissue-specific oncogenesis. By demonstrating how interfacial tension at the mutant-wild-type boundary governs whether oncogenic cells are restrained or overgrow, our work shifts the focus from purely molecular pathways to the biophysical constraints that regulate tumor initiation.

While previous studies have emphasized the contextual nature of tumorigenesis (Schneider et al., 2017; Haigis et al., 2019; Bianchi, 2020) and highlighted the critical role of interactions between healthy and oncogenic cells during epithelial cancer initiation (Hogan et al., 2009; Gupta et al., 2024; Moruzzi et al., 2021), our work integrates and extends these perspectives by demonstrating how interfacial tension at the mutant-wild-type boundary determines whether transformed cells remain constrained or undergo unchecked expansion.

Importantly, while classical frameworks based on interfacial tension have often been interpreted through the lens of the DITH (Foty and Steinberg, 2005; Brodland, 2002), which attributes segregation to global differences in the contractility and adhesive properties of two tissues displaying different intrinsic tensions, the results of the present work support a different scenario, where what counts is the difference in the heterotypic interfacial tension, i.e., the tension along the boundary of the wild-type and mutant populations. We demonstrate that this heterotypic interfacial tension is a key determinant of mutant cluster morphology and dynamics. This is also consistent with recent theoretical work showing that sharp interfaces can emerge from interfacial mechanics alone (Sussman et al., 2018). When interfacial tension between wild-type and mutant populations was positive, mutant clusters shrunk into circular aggregates and stayed constrained. In contrast, a negative interfacial tension resulted in irregular, protrusive clusters that failed to compact and kept growing. In line with this, we also showed that disrupting the interfacial actomyosin belt around the mutant clusters in the mammary epithelia disrupted the restraint on them, preventing their demixing and compaction and shifting cluster behavior to a more unjammed state, similar to the ones in the bronchial system. These findings underscore the role of interfacial cellular mechanics in shaping oncogenic cell fate, with mammary epithelium imposing physical constraints that limit mutant expansion, while bronchial epithelium lacks such mechanical barriers, permitting unchecked growth.

By integrating mechanical constraints into models of cancer initiation, our study provides a framework for understanding how different epithelial tissues resist or permit tumorigenesis. However, while interfacial tension emerges as a key regulator of oncogenic segregation, the molecular pathways driving these mechanical differences remain to be elucidated. Future studies should investigate how targeting specific signaling networks or cytoskeletal components influences interfacial tension and whether modulating these factors could provide therapeutic benefits. Additionally, extending this framework to other oncogenes and tissue types could further elucidate the mechanical principles governing tumor initiation and progression.

In conclusion, this work supports a growing view of cancer initiation as a mechanically regulated, tissue-dependent process rather than a purely mutation-driven event. By positioning interfacial mechanics between oncogenic mutant cells and their wild-type counterparts, alongside genetic and biochemical factors, our findings contribute to an emerging physical understanding of cancer, in which tissue mechanics play an active role in shaping oncogenic fate. Such a framework not only helps explain tissue-specific cancer susceptibility but also suggests that reinforcing mechanical barriers within epithelia may represent an underexplored strategy for limiting early tumor progression.

## Materials and methods

**Key resources table:** 

Reagent type (species) or resource	Designation	Source or reference	Identifiers	Additional information
Cell line (*Homo sapiens*)	MCF10A	ATCC	CRL-10317	Immortalized, non-tumorigenic human mammary epithelial cell line
Cell line (*Homo sapiens*)	BEAS2B	Gift from Arjun Guha, inSTEM, Bangalore		Immortalized, non-tumorigenic human bronchial epithelial cell line
Cell line (*Canis lupus familiari*s, female)	MDCK	Gift from Tamal Das, TIFR Hyderabad		Immortalized, non-tumorigenic, canine kidney epithelial cell line; widely used as a model for epithelial polarity and mechanics
Transfected construct (*H. sapiens* (human))	HRasV IRES GFP plasmid	Addgene; Ohoka, 2015	Plasmid #18780	Retroviral plasmid constitutively expressing along with HRas^V12^ and GFP
Antibody	E-cadherin 24E10 (Rabbit monoclonal)	Cell Signaling Technology	Cat# 3195	IF(1:1000)
Antibody	Anti-rabbit IgG (H+L), F(ab')2 Fragment (Alexa Fluor 594 Conjugate) (Rabbit polyclonal)	Cell Signaling Technology	Cat# 8889	IF(1:500)
Antibody	Phospho-Myosin Light Chain 2 (Ser19) (Rabbit polyclonal)	Cell Signaling Technology	Cat# 3671	1:50
Chemical compound, drug	Lipofectamine 3000	Thermo Fisher	Cat# L3000015	Reagent used for transfecting HRas^V12^-GFP plasmid
Peptide, recombinant protein	Fibronectin	Sigma-Aldrich	Cat# F1141	Surface coating on glass-bottom dishes
Chemical compound, drug	Blebbistatin	Sigma-Aldrich	**B0560**	Myosin II inhibitor (final concentration: 5 µM)
Software, algorithm	SPSS	SPSS	RRID:SCR_002865	
Other	DAPI stain	Cell Signaling Technology	Cat# 4083	1:1000
Other	Phalloidin stain	Cell Signaling Technology	Cat# 12877	1:300
Software, algorithm	ImageJ/Fiji	NIH	RRID:SCR_002285	Image analysis
Software, algorithm	MATLAB	MathWorks	RRID:SCR_001622	PIV (PIVlab) and Bayesian force inference analysis
Software, algorithm	Cellpose	Stringer et al., 2021.	RRID:SCR_021716	Cell segmentation
Software, algorithm	Tissue Analyser	Aigouy et al., 2016		Cell segmentation for force inference
Software, algorithm	PIVlab	Thielicke and Sonntag, 2021		Particle image velocimetry
Software, algorithm	GraphPad Prism 10	GraphPad Software	RRID:SCR_002798	Statistical analysis
Software, algorithm	Python	https://www.python.org/		Vertex model code was written in Python

### Cell line authentication

MCF10A cells were sourced from ATCC and were authenticated at the source. BEAS2B cells were a gift from Arjun Guha, and MDCK cells were a gift from Tamal Das. MDCK cells were originally sourced from the European Collection of Authenticated Cell Cultures and were authenticated at the source. BEAS2B cells were originally sourced from ATCC and were authenticated at the source.

Cell lines used in the study were free of mycoplasma contamination.

### Cell culture

Non-transformed human mammary epithelial cell line MCF10A was maintained in complete medium composed of phenol-free Dulbecco’s modified Eagle’s medium (DMEM/F-12, Gibco) supplemented with 5% charcoal-stripped horse serum (Gibco), 10 U/ml penicillin and 10 μg/ml streptomycin (Pen-Strep, Invitrogen), epidermal growth factor (20 ng/ml; PeproTech), hydrocortisone (0.5 mg/ml; Sigma-Aldrich), cholera toxin (100 ng/ml; Sigma-Aldrich), and insulin (10 μg/ml; Sigma-Aldrich) at 37°C in a humidified incubator with 5% CO_2_.

Non-transformed human bronchial epithelium epithelial cell line BEAS2B was cultured in DMEM (Gibco) supplemented with GlutaMAX (Gibco) and 5% fetal bovine serum (FBS, Gibco) along with 10 U/ml penicillin and 10 μg/ml streptomycin (Pen-Strep, Invitrogen). Cells were maintained at 37°C and 5% CO_2_ unless mentioned otherwise.

### Cell seeding

10^5^ Cells were seeded on fibronectin-coated (10 μg/ml) glass-bottom dishes (35 mm Cellvis) and transfected once they reached 80% confluency (for singlets) and 60% confluency to get transfected groups.

### Transfection

Cells were transiently transfected with the Hras^V12^-GFP expressing plasmid DNA (Addgene #18780) using Lipofectamine 3000 (Invitrogen), following the manufacturer’s protocol. Post-transfection, confluent monolayers were either fixed and immunostained or imaged directly.

### Blebbistatin addition

Blebbistatin was diluted to 5 μM final solution and added 48 hr post-transfection. Samples were washed after 24 hr and fixed right away.

### Immunofluorescence

Cells were fixed with 4% paraformaldehyde (Thermo Fisher) diluted in 1× phosphate-buffered saline (PBS, Sigma-Aldrich) for 15 min at room temperature. After washing away the fixative with 1× PBS, cells were permeabilized with 0.3% Triton X-100 (Sigma-Aldrich) in 1× PBS. Nonspecific antibody binding was blocked by incubating the samples in blocking solution (2% bovine serum albumin [BSA] Sigma-Aldrich and 2% FBS in PBS) at room temperature for 45 min. Further, cells were incubated with primary antibody prepared in blocking/staining solution overnight at 4°C. Post primary antibodies incubation, cells were washed thrice with 1× PBS for 5 min per wash and incubated with secondary antibodies conjugated to Alexa Fluor 555, Alexa Fluor 594-conjugated phalloidin, and 4′,6-diamidino-2-phenylindole (DAPI) to mark the cell nucleus, prepared in blocking/staining solution, for 2 hr at room temperature. Finally, the samples were washed four times with 1× PBS for 5 min per wash before proceeding to microscopy.

### Microscopy

Fluorescence images were acquired using a 20× objective and a 63× oil-immersion objective of a Zeiss Axio Observer 7 Inverted Microscope with a scientific-grade sCMOS camera by Iberoptics.

For live imaging, samples were set up on a stage-top incubator and maintained at 37°C and 5% CO_2_ throughout imaging.

### Image analysis

Image analysis for this study was performed using Fiji (Schindelin et al., 2012) except Bayesian force inference and PIV analysis, which were performed in MATLAB (MathWorks). Cellpose (Stringer et al., 2021) was used to segment cells to get cell count, shape indices, and actin intensities for each cell while Tissue Analyzer (Aigouy et al., 2016) was used to segment cells for force inference.

### Quantification of cluster eccentricity and segregation index

The term ‘eccentricity’ expresses a size reduction in the contact with neighboring cells. It was quantified using the formula $e=\sqrt{\left (1-\left (\frac{b}{a}\right)^{2}\right)}$ (where *a*=the length of the major axes and *b*=the length of the minor axes of the clusters), with a value closer to 0 indicating a more circular shape. Eccentricity quantifications were performed on fixed and live tissues using outlines that were manually drawn and measured using Fiji.

The segregation index SI, defined as the average ratio of homotypic and all cell neighbors as done previously (Skamrahl et al., 2023), quantifies the demixing degree. To quantify the SI, cells of each type were manually counted, and finally, to generate the plots, the SI was averaged over all GFP-labeled cells in one frame.

### Bayesian force inference

Bayesian force inference was done as described previously (Ishihara and Sugimura, 2012) to determine the relative cellular pressures and cell-cell edge or line tension.

### Particle image velocimetry

PIV was done using the PIVlab package on MATLAB (Thielicke and Sonntag, 2021). FFT window deformation (direct Fourier transform correlation with multiple passes and deforming windows) algorithm was used. Velocity maps were obtained by a three-pass interrogation window of 128×128 pixels that halved after each pass. Tangential velocities were obtained by drawing concentric circles radially outward from the center to a point at the boundary of the mutant clusters and integrated over the entire circle. The output is the tangential velocity as a function of the circle.

### Statistical analysis

Statistical analyses were carried out in GraphPad Prism 10. Statistical significance was calculated by an unpaired t-test with Welch’s correction. p-Values greater than 0.05 were considered to be statistically not significant. Each plot has data from one out of three independent experiments. Violin plots have the median shown as a bold dashed line and the first and third quartiles shown as thin dashed lines. Box and whisker plots have whiskers extending from the minimum to the maximum values, showing the full range of the dataset, including outliers. No statistical methods were used to set the sample size.

### Vertex model

A bi-disperse model is used to demonstrate the role of interfacial tension between two types of cells, specifically the HRas^V12^ mutant and wild-type cells.

$$\displaystyle  E_{VM} = &  \sum_{\alpha \in \mathrm{WT}} \left[ \frac{K_{\alpha}^{\mathrm{WT}}}{2} \left(A_{\alpha}-A_{\alpha}^{0,\mathrm{WT}}\right)^{2} + \frac{\Gamma_{\alpha}^{\mathrm{WT}}}{2} \left(P_{\alpha}-P_{\alpha}^{0,\mathrm{WT}}\right)^{2} \right] +\\ &  \sum_{\alpha \in \mathrm{M}} \left[ \frac{K_{\alpha}^{\mathrm{M}}}{2} \left(A_{\alpha}-A_{\alpha}^{0,\mathrm{M}}\right)^{2} + \frac{\Gamma_{\alpha}^{\mathrm{M}}}{2} \left(P_{\alpha}-P_{\alpha}^{0,\mathrm{M}}\right)^{2} \right] + \sum_{\langle i,j\rangle} \Lambda_{ij}\,l_{ij}$$

The first two sums are included in the standard vertex model, whereas the last term is introduced in our model to account for interfacial interactions between cells. Specifically, $\Lambda _{ij}=0$ for interfaces between same cell types, and $\Lambda _{ij}=\Lambda $ between different cell types. Using a line tension term such as $\Lambda l_{ij}$ in the vertex model energy, where the $l_{ij}$ is the length of an interface edge, we can obtain both segregation and spreading dynamics just from energy minimization. This term has been described as heterotypic line tension (Canty et al., 2017). Due to energy minimization, heterotypic interface length is minimized for $\Lambda > 0$ and maximized for $\Lambda < 0$, leading to segregation of the two cell types in the former case and complete mixing of them in the latter case.

The mutant cluster in the experiments with MCF10A cells showed an actin belt forming along the cluster interface, with the cells along the interface being more elongated than normal. This actin ring is important for the compaction of mutant clusters in the presence of wild-type cells in the epithelial monolayer. An isotropic interfacial tension, such as the $\Lambda l_{ij}$ term in our model, cannot capture such anisotropic behavior. Recently, it has been proposed that such anisotropic behavior can arise from the anisotropic distribution of the cytoskeletal filaments inside the cell (Moruzzi et al., 2021). The resulting active interfacial tension, called the shape-tension coupling, can generate elongated shapes as observed in the experiments. The elongated cells in the experiments indeed have anisotropic distributions of the stress fibers. Hence, we included the effect of shape-tension in our model. The total interfacial tension is $\Lambda _{ij}+\gamma _{ij}\left (t;\Pi _{0}\right)$, where $\Lambda _{ij}$ is a time-independent, passive, isotropic tension, and $\gamma _{ij}$ is a time-dependent active tension that depends on the shape of the cells sharing the interface and requires a threshold stress $\Pi _{0}$ to be non-zero. The details of the shape-tension coupling are described in Appendix 1. We find that a non-zero activation stress $\Pi _{0}$ is necessary to reproduce experimentally observed results.

## Figures and Tables

**Appendix 1—figure 1.**
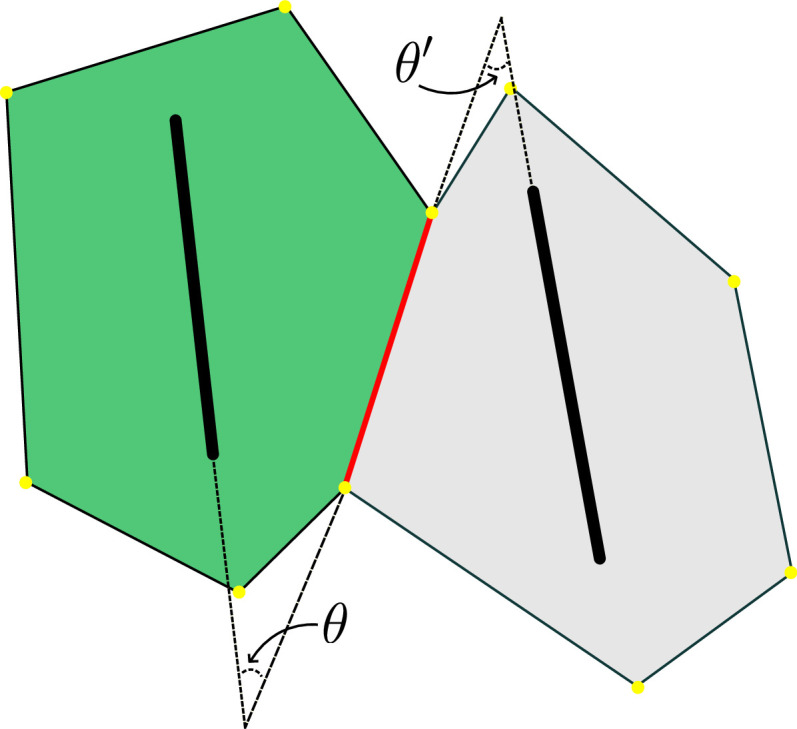
Illustration of shape-tension coupling at the mutant (green)-wild-type (gray) interface (red), where angles *θ* and *θ*’ represent the orientation of boundary cells relative to their shared edge.

**Appendix 1—figure 2.**
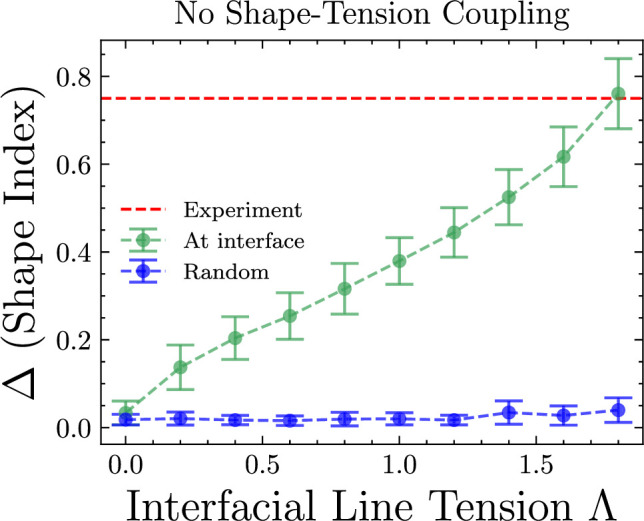
Shape indices vs. the interfacial line tension for no shape-tension coupling.

**Appendix 1—figure 3.**
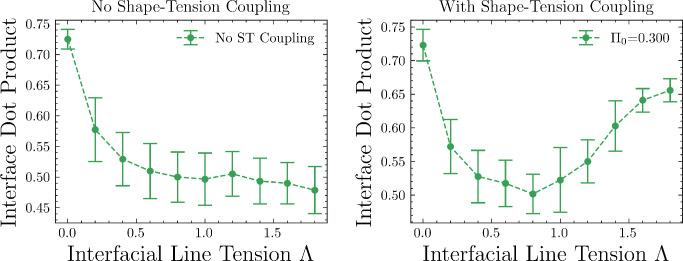
Interface dot product for different interfacial line tension with and without shape-tension coupling.

**Appendix 1—figure 4.**
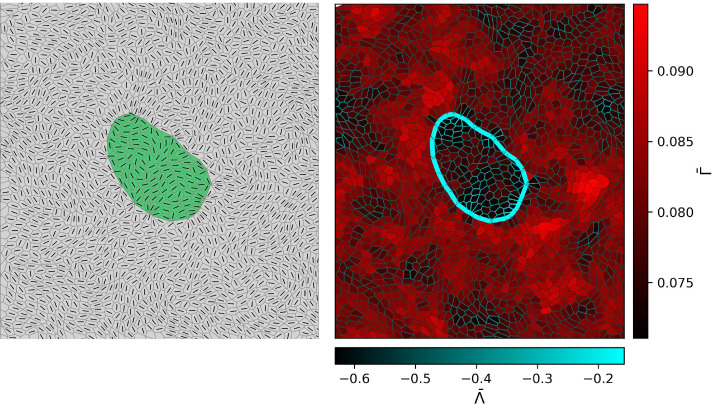
For positive differential interfacial tension (Λ >0), the mutant cluster has lower bulk actin level compared to the wild-type cells surrounding it.

**Appendix 1—figure 5.**
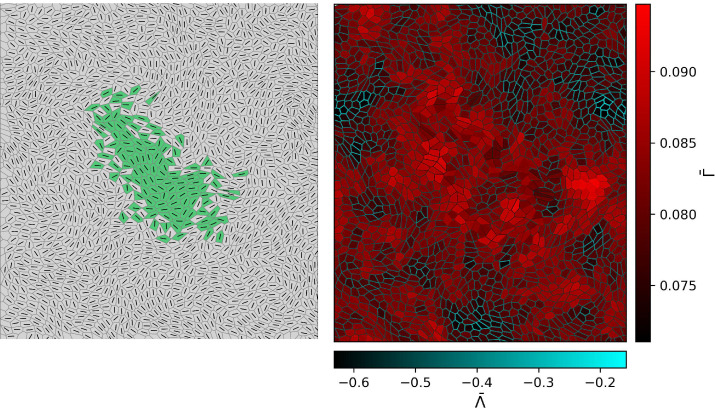
For negative differential interfacial tension (Λ<0), the mutant cluster does not have a very different actin level compared to the wild-type cells surrounding it.

**Appendix 1—figure 6.**
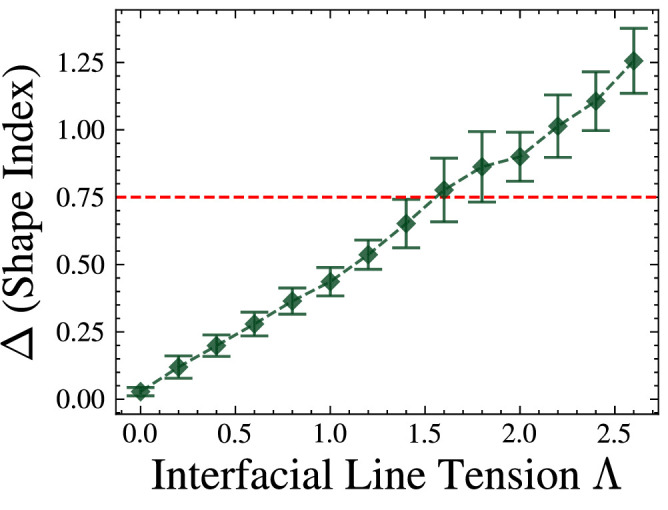
Difference in shape index between interfacial and bulk cells as a function of Λ (interfacialt ension) for the shear stress-activated model.

## Data Availability

All imaging data supporting the main findings of this study, including immunostaining and live-imaging data, have been deposited in the Zenodo repository (https://doi.org/10.5281/zenodo.21801094). All the source data files are available at https://github.com/Amrapali27/elifepaper and all the codes used in this study are available at https://github.com/SSarkarGroup/Active_VM_MCFL (copy archived at Dewan, 2026). The following datasets were generated: DattaA 2026Heterotypic interfacial tension between oncogenic and wild-type populations forms the mechanical basis of tissue-specific oncogenesis in epitheliaGitHubAmrapali27/elifepaper10.7554/eLife.106893PMC1344690342559715 DattaA VishwakarmaM 2026Heterotypic interfacial tension between oncogenic and wild-type populations forms the mechanical basis of tissue-specific oncogenesis in epitheliaZenodo10.5281/zenodo.21801094PMC1344690342559715

## Additional files

MDAR checklist

## Appendix 1

### Vertex model simulation

To understand the cause and the dynamics of interaction between mutant and wild-type populations in different epithelial monolayers during cancer initiation, we used a cell-based model of epithelial tissues called the vertex model. A monolayer in the vertex model has total energy given by:

$$\displaystyle  E_{VM}=\sum _{\alpha }\left [\frac{K_{\alpha }}{2}\left (A_{\alpha }-A_{\alpha }^{0}\right)^{2}+\frac{\Gamma _{\alpha }}{2}\left (P_{\alpha }-P_{\alpha }^{0}\right)^{2}\right ]$$

In the above energy function, the constant parameters $K_{\alpha }$ are the area modulus, which gives the area elasticity of each cell, and $\Gamma _{\alpha }$ is the perimeter modulus, which gives the elasticity due to the actomyosin cortex. The gradient of this energy function gives the forces on each vertex of the cell, $\overset{\rightarrow }{F}^{\left (i\right)}=-\nabla _{\overset{\rightarrow }{r}^{\left (i\right)}}E_{VM}$, where $\overset{\rightarrow }{r}^{\left (i\right)}{\ }$ is the position of the *i*th vertex. The dynamics of each vertex position is given by the overdamped equation of motion:

$$\displaystyle \frac{d\overset{\rightarrow }{r}^{\left (i\right)}}{dt}=\overset{\rightarrow }{F}^{\left (i\right)}+\overset{\rightarrow }{F}_{act}^{\left (i\right)}+\overset{\rightarrow }{F}_{motility}^{\left (i\right)}$$

where $\overset{\rightarrow }{F}_{act}^{\left (i\right)}$ is any active force that might drive the system out of equilibrium. $\overset{\rightarrow }{F}_{motility}^{\left (i\right)}$ is a force that propels individual cells by $\overset{\rightarrow }{F}_{motility}^{\left (i\right)}=v_{0}\overset{\rightarrow }{p}_{\alpha }$, where $\overset{\rightarrow }{p}_{\alpha }$ is the polarity of a given cell given by $\overset{\rightarrow }{p}_{\alpha }=\left (\cos\theta _{\alpha },\sin\theta _{\alpha }\right)$, where this angle $\theta _{\alpha }$ performs rotational diffusion:

$$\displaystyle \partial _{t}\theta _{\alpha }=\sqrt{2D_{r}}\eta _{\alpha }\left (t\right)$$

where $\eta _{\alpha }\left (t\right)$ is a Gaussian white noise with zero mean and correlation $\left \langle \eta _{\alpha }\left (t\right)\eta _{\alpha ^{'}}\left (t^{'}\right)\right \rangle =\delta \left (t-t^{'}\right)\delta _{\alpha \alpha ^{'}}$.

To capture the dynamics of cell segregation between two different types of cells in a monolayer of normal and oncogenic cells, we can use a bi-disperse vertex model with an interfacial line tension between the two types of cells, mutant and wild-type.

The director of each cell corresponds to the polarization direction of the cell, which can be obtained as the larger eigenvector of its shape tensor, $\underline{\underline{G_\alpha}}=\frac{1}{N^{(\alpha)}} \sum_{i \in v_\alpha}\left(\vec{r}^{(i)}-\vec{r}_0^{(\alpha)}\right) \otimes\left(\vec{r}^{(i)}-\vec{r}_0^{(\alpha)}\right)$, where the sum is over $N^{\left (\alpha \right)}$ vertices of the cell α, and $\overset{\rightarrow }{r}_{0}^{\left (\alpha \right)}$ is the position of the cell’s geometric center. The energy function for the vertex model used is given below:

$$\displaystyle   E_{VM} = & \sum_{\alpha \in \mathrm{WT}} \left[ \frac{K_{\alpha}^{\mathrm{WT}}}{2} \left(A_{\alpha}-A_{\alpha}^{0,\mathrm{WT}}\right)^{2} + \frac{\Gamma_{\alpha}^{\mathrm{WT}}}{2} \left(P_{\alpha}-P_{\alpha}^{0,\mathrm{WT}}\right)^{2} \right] + \\ & \sum_{\alpha \in \mathrm{M}} \left[ \frac{K_{\alpha}^{\mathrm{M}}}{2} \left(A_{\alpha}-A_{\alpha}^{0,\mathrm{M}}\right)^{2} + \frac{\Gamma_{\alpha}^{\mathrm{M}}}{2} \left(P_{\alpha}-P_{\alpha}^{0,\mathrm{M}}\right)^{2} \right] + \sum_{\langle i,j\rangle} \Lambda_{ij}\,l_{ij}$$

Here, $M$ denotes mutant cells and $WT$ denotes wild-type cells. The index $\alpha $ denotes cells, and the indices $i,j$ denote vertices and ${< }ij{> }$ denotes an edge between vertices $i$ and $j$. The parameters $K_{\alpha }^{M}$, $K_{\alpha }^{WT}$ are the area moduli, $\Gamma _{\alpha }^{M}$, $\Gamma _{\alpha }^{WT}$ are the perimeter moduli of the mutant and wild-type, respectively, and the $\Lambda _{ij}$ denotes the interfacial line tension. This interfacial line tension is only along junctions connecting two vertices and depends on the cell type on either side of the junction. Since the system we are considering is bi-disperse, the interfacial line tension can have three values, depending on the identities of cells on either side of a given edge $< ij> $:

$$\displaystyle \Lambda_{ij} = \begin{cases} \qquad \Lambda_{ij}^{\mathrm{M}/\mathrm{M}} & \text{if both are mutant cells} \\[4pt] \quad \Lambda_{ij}^{\mathrm{WT}/\mathrm{WT}} & \text{if both are wild-type cells} \\[4pt] \Lambda_{ij}^{\mathrm{WT}/\mathrm{M}} & \text{if one is wild-type and the other is mutant} \end{cases}$$

### Shape-tension coupling

In addition to this passive interfacial tension, we can also have an active contribution coming from the shape-tension coupling. The shape-tension coupling is mathematically the same as the passive line tension force. The total force is given by:

$$\displaystyle \left (\Lambda +\gamma _{ij}\left (t\right)\right)\overset{\rightarrow }{\nabla }l_{ij}$$

$\gamma _{ij}\left (t\right){\ }$ depends on the relative orientation of the cell’s director $\overset{\rightarrow }{n}$ and the edge along which the tension acts, $\overset{\rightarrow }{l}_{ij}$. Specifically, it has a relaxational dynamics of the form:

$$\displaystyle \dot{\gamma}_{ij} =[{\gamma_{ij}(t)-\gamma_{ij,0}}]/{\tau}.$$

Here, $\gamma_{ij,0} = -\frac{\zeta}{2} \left[ \cos(2\theta) + \cos(2\theta') \right]$ couples the tension along an edge to the shape of the cells on either side of the edge (Appendix 1—figure 1).

For $\zeta > 0$, this tension is extensile and leads to the elongation of cells along the boundary. This happens because for the edges that have $\overset{\rightarrow }{n}$ and $\overset{\rightarrow }{n}'$ aligned with the edge vector ($\theta \text{ or } \theta' = 0,\ \pi,\ 2\pi$), the shape-tension coupling contributes a negative line tension, which favors this alignment. The directors can be obtained from the larger eigenvector of the shape tensor of the cells as described before. When the edge vectors are not aligned with the edge vector, the contribution is positive, and that edge starts to shrink. Eventually, the interface consists of cells whose directors are always aligned with the interface. Energetically, it counteracts interfaces with $\Lambda > 0$, leading to the formation of elongated cells and tangential flows along the interface. Importantly, we find that cell elongation correlates with the formation of an actin belt, leading us to postulate that the shape-tension coupling is activated above a threshold pressure, $\Pi _{0}$, where the cell pressure is given by $\Pi =-\frac{\partial E_{VM}}{\partial A}$. When $\Lambda < 0$, such as in the BEAS2B cells, we do not observe strong stress-fiber expression. Hence, we believe that it is not activated in those cells.

### Simulation setup

The vertex model energy can be non-dimensionalized by setting a length scale set by $\sqrt{A^{0}}$. In our simulation, we have taken

$$\displaystyle A_{\alpha}^{0,\mathrm{WT}} = A_{\alpha}^{0,\mathrm{M}} = A^{0},\, \forall\,\alpha$$

and

$$\displaystyle P_{\alpha}^{0,\mathrm{WT}} = P_{\alpha}^{0,\mathrm{M}} = P^{0} \, \forall\,\alpha.$$

Also,

$$\displaystyle K_{\alpha}^{\mathrm{WT}} = K_{\alpha}^{\mathrm{M}} = K \, \forall\,\alpha,$$

$$\displaystyle \Gamma_{\alpha}^{\mathrm{WT}} = \Gamma_{\alpha}^{\mathrm{M}} = \Gamma \, \forall\,\alpha$$

and

$$\displaystyle \Lambda_{ij}^{\mathrm{M}/\mathrm{M}} = \Lambda_{ij}^{\mathrm{WT}/\mathrm{WT}} = 0, \quad \forall\,\langle i,j\rangle, \Lambda_{ij}^{\mathrm{M}/\mathrm{WT}} = \Lambda \, \forall\,\langle i,j\rangle.$$

The non-dimensionalization has been done, which defines the normalized parameters $\bar{\Gamma }=\Gamma KA^{0}\,\bar {\Lambda }=\Lambda K\left (A^{0}\right)^{3\mathrm{/}2}$. (In further discussions, the normalized parameters will be represented by the symbols $\Gamma $ and $\Lambda $.)

The monolayer modeled by the vertex model can be described by a single number, the shape index, defined by $q=P^{0}/\sqrt{A^{0}}$. Here too, in principle, we could have different shape indices for the mutant and wild-type cells, but we take them to be the same. Unless otherwise mentioned, we take $\Lambda ^{M/M}=\Lambda ^{WT/WT}=0$ and $\Lambda ^{WT/M}=\Lambda $.

To simulate the elongation of cells along the interface, $\zeta> 0$ is used for an active extensile contribution to the interfacial tension.

A box containing 20×20 cells has been used with mutant cells placed within a region (non-circular) from the center of the box. The vertices are arranged in a hexagonal lattice with random displacements added to the position of each vertex. Periodic boundary conditions were used to simulate a confluent monolayer.

### Parameters

**Table:**

Parameter	Value
*K*	1
Γ	0.1
*A* ^0^	1
*P* ^0^	4
*l*_*min*_ for T1 transitions	0.04
Δ*t* for time stepping	0.01
*v*_0_ for motility	0.05
*D*_*r*_ for rotational diffusion	0.5
ζ for shape-tension coupling	0.6

To prevent overlaps, a node switch operation is also added to the simulation, which changes the topology of the network (Fletcher et al., 2013).

### Extrusion probability calculation

Simulations with just a single mutant cell were run for a range of heterotypic interfacial line tension values ($\Lambda \mathrm{=}{\ }0,{\ }0.1,{\ }0.4,{\ }0.8,{\ }1.2,{\ }1.6$) with shape-tension coupling. The simulation was run until the area of the mutant cell fell below a threshold area =0.1, after which we consider the mutant cell to be extruded. 9 different random initial seeds were run and analyzed. Each seed gives a binary result – either extruded or not. This was used to calculate the extrusion probability.

### Need for shape-tension coupling – elongation along interface

Shape-tension coupling has been used here in accordance with the experimental observation that the cells at the interface are aligned and elongated along the interface (Figure 2h). We plotted the difference between shape indices of cells at the interface and away from the boundary vs. the interfacial tension in the case of no shape-tension coupling (Appendix 1—figure 2). The red dashed line represents the experimental value of the shape index difference. The blue line is the shape index difference between two randomly chosen groups of cells (half of the total number of cells in each group is taken). At zero line tension, the difference in shape index between interface cells and cells away from the interface is the same as that between randomly chosen groups of cells, which is expected since there should be no interface at zero line tension. The no shape-tension data presented here are averaged over 19 seeds. Although the results without shape-tension coupling reach experimental values at high enough interfacial tension (Appendix 1—figure 2), a closer inspection of the simulation results shows that the cells are just squeezed and are aligned perpendicular to the interface, which is contrary to what is seen in experiments (Figure 2h).

Calculating the average of the absolute value of the dot product of the nematic director and the interface edge for simulations with and without shape-tension coupling (Appendix 1—figure 3) clearly shows that with shape-tension coupling, the cells align and elongate along the interface as is seen in experiment, given by an interface dot product value >0.5 at high enough line-tension values. Further, shape-tension coupling or biased edge tension has been used before to model for cell elongation during embryo elongation (Dye et al., 2021), and here we use it as an active line-tension force, which elongates cells along the interface, in addition to the interfacial tension, which is passive.

### Vertex model with mechanochemical feedback

Although the difference in actin between mutant and wild-type has not been incorporated in the model presented in the manuscript, we could see how the actin levels change in response to the interfacial tension formed between the mutant and wild-type cells by adding a mechanochemical feedback in the model. As given in Muthukrishnan et al., 2025, we can model the behavior of tissues using a vertex model with mechanochemical feedback. It has been shown that incorporating these feedback loops in the vertex model captures the biologically realistic features of epithelial tissues seen in experiment (Muthukrishnan et al., 2025). In particular, to model the actin distribution in monolayers with mutants and wild-type, we can use a vertex model with interfacial tension and MCFL-I (Muthukrishnan et al., 2025). When we incorporate MCFL-I in our model, we can observe how the normalized contractility $\bar{\Gamma }=\left (1/K\right)\left (1/A_{0}\right)$ and normalized line tension $\bar{\Lambda }=-\left (\Gamma /K\right)\left (P_{0}/A_{0}^{3/2}\right)$ change with different line tensions. The normalized contractility is associated with the bulk actin or the bulk compressibility, and the normalized line tension is associated with the junctional actin of the cells. The equations for MCFL-I can be written as:

$$\displaystyle \tau_{A}\,\dot{A}_{0} = -\left[ A_{0} - \hat{A}_{0} \right]$$

$$\displaystyle \tau_{P}\,\dot{P}_{0} = -\left[ P_{0} - \hat{P}_{0} \right]$$

Thus, with MCFLs, the vertex model does not have fixed $A_{0}$ and $P_{0}$. The cells dynamically change these parameters depending on the vertex model dynamics. The constitutive relations for the $\hat{A}_{0}$ and $\hat{P}_{0}$ are given below (Muthukrishnan et al., 2025):

$$\displaystyle \hat {A}_{0}=2a_{0}p_{bound}$$

$$\displaystyle \hat {P}_{0}\mathrm{=}2q_{0}\sqrt{a_{0}}\left (1\mathrm{-}p_{bound}\right)$$

Here, $p_{bound}=1/\left (1+\left (A/\hat {A}\right)^{2}\right)$, which is the probability of myosin binding and $\hat {A}$ is a model parameter which depends on the dissociation constant of myosin (Muthukrishnan et al., 2025). We consider $\hat{A}$ to be the same for both mutant and wild-type ($\hat {A}=1.3$). A positive heterotypic interfacial tension can lead to compression (decrease in area) of the mutants, whereas a negative heterotypic interfacial tension can lead to relaxation of cell area of the mutants. This will lead to different $A_{0}$ and $P_{0}$ across the monolayer, and thus different $\bar{\Gamma }$ and $\bar{\Lambda }$, which provides an understanding of the different actin levels.

For positive heterotypic interfacial tension, the mutants are compressed, which leads to a decrease in $\bar{\Gamma }$ and an increase in $\bar{\Lambda }$ (Appendix 1—figure 4). In the figure, we plot the $\bar{\Gamma }$ as the bulk actin and the $\bar{\Lambda }$ as the junctional actin. Since our model has an active line tension, the normalized line tension of an edge will also have a contribution from $\Lambda $, thus $\bar{\Lambda }=-\left (\Gamma /K\right)\left (P_{0}/A_{0}^{3/2}\right)+\left (1/K\right)\left (\Lambda /A_{0}^{3/2}\right)$. The spatial distribution of actin in the mutant and wild-type seen in experiments matches well with the snapshot from simulations, where for positive heterotypic interfacial tension, the bulk actin is low within the cluster as compared to the wild-type (Appendix 1—figure 4). For negative heterotypic interfacial tension too, we see that the actin levels are not very different between the mutant cluster and the wild-type (Appendix 1—figure 5).

### Stress-activated elongation

In our model, the active tension responsible for cell elongation along the interface is activated via isotropic pressure and can be compactly represented as:

$$\displaystyle \gamma_{ij}^{0} = \frac{\zeta}{2} \left[ \frac{1}{{tr}\,\underline{\underline{G}}}\, \hat{t}_{ij}\cdot\tilde{\underline{\underline{G}}}\cdot\hat{t}_{ij} + \frac{1}{{tr}\,\underline{\underline{G}}'}\, \hat{t}_{ij}\cdot\tilde{\underline{\underline{G}}}'\cdot\hat{t}_{ij} \right] \Theta\!\left(\Pi\gt\Pi_{0}\right)$$

where $\hat {t}_{ij}$ is the unit vector tangent to the edge $ij$ and $\overset{\sim }{G}$ is the anisotropic part of the shape tensor, with $\frac{1}{tr\, {\underline{\underline{G}}}}\hat {t}_{ij}.\overset{\sim }{\underline{\underline{G}}}.\hat {t}_{ij}\mathrm{=}cos2\theta ,$ and $\frac{1}{tr\, {\underline{\underline{G'}}}}\hat {t}_{ij}.\overset{\sim }{\underline{\underline{G'}}}.\hat {t}_{ij}\mathrm{=}cos2\theta '$. This active tension is coupled to the cell shape and is only activated when the isotropic pressure is above a certain threshold, $\Pi _{0}$, which is a model parameter. This is taken to be a proxy for the strength of heterotypic interactions at the interface. This model represents the following feedback which we postulate: isotropic pressure (proxy) → active tension → cell elongation. However, we can also write a model in which this is activated not by the isotropic pressure but by the shear stress on the cells at the interface. Then, the active tension can be written as:

$$\displaystyle \gamma _{ij}^{0}=\frac{\zeta }{2}\left [\hat {t}_{ij}.\overset{\sim }{{\underline{\underline{\sigma}}}}.\hat {t}_{ij}\mathrm{+}\hat {t}_{ij}.\overset{\sim }{{\underline{\underline{\sigma}}}'}.\hat {t}_{ij}\right ]$$

where $\overset{\sim }{\sigma }$ is shear stress related to the stress tensor as $\overset{\sim }{\sigma }=\sigma \mathrm{-}\frac{1}{2}tr\left (\sigma \right)I$. The stress tensor of a cell $\alpha $ is written as (Yang et al., 2017): $\sigma _{ab}^{\alpha }=-\Pi _{\alpha }\delta _{ab}+\frac{1}{2 A}\sum _{ij\in \alpha }T_{ij}^{a}l_{ij}^{b}$, with $\Pi _{\alpha }=-\frac{\partial E}{\partial A_{\alpha }},\overset{\rightarrow }{T}_{ij}=\frac{\partial E}{\partial \overset{\rightarrow }{l}_{ij}}=T_{ij}\hat{l}_{ij}$. This is a direct coupling between the active tension and the shear stress. Note that the model parameter $\Pi _{0}$ is not needed anymore. This modification is similar to the mechanosensitive feedback given in Pérez-Verdugo et al., 2026: interfacial tension → shear stress → active tension → cell elongation → shear stress.

Both models give similar cell shapes at the interface in simulations (Appendix 1—figure 6).

## References

[bib1] Aigouy B, Umetsu D, Eaton S (2016). Segmentation and quantitative analysis of epithelial tissues. Drosophila: Methods and Protocols.

[bib2] Alt S, Ganguly P, Salbreux G (2017). Vertex models: from cell mechanics to tissue morphogenesis. Philosophical Transactions of the Royal Society of London. Series B, Biological Sciences.

[bib3] Atia L, Bi D, Sharma Y, Mitchel JA, Gweon B, Koehler SA, Fredberg JJ (2018). Geometric constraints during epithelial jamming. Nature Physics.

[bib4] Barton DL, Henkes S, Weijer CJ, Sknepnek R (2017). ACtive vertex model for cell-resolution description of epithelial tissue mechanics. PLOS Computational Biology.

[bib5] Beroukhim R, Mermel CH, Porter D, Wei G, Raychaudhuri S, Donovan J, Barretina J, Boehm JS, Dobson J, Urashima M, Mc Henry KT, Pinchback RM, Ligon AH, Cho YJ, Haery L, Greulich H, Reich M, Winckler W, Lawrence MS, Weir BA, Tanaka KE, Chiang DY, Bass AJ, Loo A, Hoffman C, Prensner J, Liefeld T, Gao Q, Yecies D, Signoretti S, Maher E, Kaye FJ, Sasaki H, Tepper JE, Fletcher JA, Tabernero J, Baselga J, Tsao MS, Demichelis F, Rubin MA, Janne PA, Daly MJ, Nucera C, Levine RL, Ebert BL, Gabriel S, Rustgi AK, Antonescu CR, Ladanyi M, Letai A, Garraway LA, Loda M, Beer DG, True LD, Okamoto A, Pomeroy SL, Singer S, Golub TR, Lander ES, Getz G, Sellers WR, Meyerson M (2010). The landscape of somatic copy-number alteration across human cancers. Nature.

[bib6] Bianchi JJ (2020). Not all cancers are created equal: tissue specificity in cancer genes and pathways. Current Opinion in Cell Biology.

[bib7] Brodland GW (2002). The differential interfacial tension hypothesis (DITH): a comprehensive theory for the self-rearrangement of embryonic cells and tissues. Journal of Biomechanical Engineering.

[bib8] Canty L, Zarour E, Kashkooli L, François P, Fagotto F (2017). Sorting at embryonic boundaries requires high heterotypic interfacial tension. Nature Communications.

[bib9] Curtius K, Wright NA, Graham TA (2018). An evolutionary perspective on field cancerization. Nature Reviews. Cancer.

[bib10] Davoli T, Xu AW, Mengwasser KE, Sack LM, Yoon JC, Park PJ, Elledge SJ (2013). Cumulative haploinsufficiency and triplosensitivity drive aneuploidy patterns and shape the cancer genome. Cell.

[bib11] Dewan P (2026). Software Heritage.

[bib12] Dye NA, Popović M, Iyer KV, Fuhrmann JF, Piscitello-Gómez R, Eaton S, Jülicher F (2021). Self-organized patterning of cell morphology via mechanosensitive feedback. eLife.

[bib13] Fletcher AG, Osborne JM, Maini PK, Gavaghan DJ (2013). Implementing vertex dynamics models of cell populations in biology within a consistent computational framework. Progress in Biophysics and Molecular Biology.

[bib14] Fletcher AG (2014). Vertex models of epithelial morphogenesis. Biophysical Journal.

[bib15] Foty RA, Steinberg MS (2005). The differential adhesion hypothesis: a direct evaluation. Developmental Biology.

[bib16] Franklin WA, Gazdar AF, Haney J, Wistuba II, La Rosa FG, Kennedy T, Ritchey DM, Miller YE (1997). Widely dispersed p53 mutation in respiratory epithelium: A novel mechanism for field carcinogenesis. The Journal of Clinical Investigation.

[bib17] Gupta P, Kayal S, Tanimura N, Pothapragada SP, Senapati HK, Devendran P, Fujita Y, Bi D, Das T (2024). Mechanical imbalance between normal and transformed cells drives epithelial homeostasis through cell competition. bioRxiv.

[bib18] Haigis KM, Cichowski K, Elledge SJ (2019). Tissue-specificity in cancer: The rule, not the exception. Science.

[bib19] Hanahan D, Weinberg RA (2000). The hallmarks of cancer. Cell.

[bib20] Hogan C, Dupré-Crochet S, Norman M, Kajita M, Zimmermann C, Pelling AE, Piddini E, Baena-López LA, Vincent JP, Itoh Y, Hosoya H, Pichaud F, Fujita Y (2009). Characterization of the interface between normal and transformed epithelial cells. Nature Cell Biology.

[bib21] Ishihara S, Sugimura K (2012). Bayesian inference of force dynamics during morphogenesis. Journal of Theoretical Biology.

[bib22] Kajita M, Sugimura K, Ohoka A, Burden J, Suganuma H, Ikegawa M, Shimada T, Kitamura T, Shindoh M, Ishikawa S, Yamamoto S, Saitoh S, Yako Y, Takahashi R, Okajima T, Kikuta J, Maijima Y, Ishii M, Tada M, Fujita Y (2014). Filamin acts as a key regulator in epithelial defence against transformed cells. Nature Communications.

[bib23] Kajita M, Fujita Y (2015). EDAC: Epithelial defence against cancer—cell competition between normal and transformed epithelial cells in mammals. Journal of Biochemistry.

[bib24] Lehmann B, Biburger M, Brückner C, Ipsen-Escobedo A, Gordan S, Lehmann C, Voehringer D, Winkler T, Schaft N, Dudziak D, Sirbu H, Weber GF, Nimmerjahn F (2017). Tumor location determines tissue-specific recruitment of tumor-associated macrophages and antibody-dependent immunotherapy response. Science Immunology.

[bib25] Leung CT, Brugge JS (2012). Outgrowth of single oncogene-expressing cells from suppressive epithelial environments. Nature.

[bib26] Manning ML, Foty RA, Steinberg MS, Schoetz EM (2010). Coaction of intercellular adhesion and cortical tension specifies tissue surface tension. PNAS.

[bib27] Martin AC (2010). Pulsation and stabilization: contractile forces that underlie morphogenesis. Developmental Biology.

[bib28] Martincorena I, Campbell PJ (2015). Somatic mutation in cancer and normal cells. Science.

[bib29] Mitchel JA, Das A, O’Sullivan MJ, Stancil IT, DeCamp SJ, Koehler S, Ocaña OH, Butler JP, Fredberg JJ, Nieto MA, Bi D, Park J-A (2020). In primary airway epithelial cells, the unjamming transition is distinct from the epithelial-to-mesenchymal transition. Nature Communications.

[bib30] Moruzzi M, Nestor-Bergmann A, Goddard GK, Tarannum N, Brennan K, Woolner S (2021). Generation of anisotropic strain dysregulates wild-type cell division at the interface between host and oncogenic tissue. Current Biology.

[bib31] Muthukrishnan S, Dewan P, Tejaswi T, Sebastian MB, Chhabra T, Mondal S, Kolya S, Sarkar S, Vishwakarma M (2025). Glassy dynamics in active epithelia emerge from an interplay of mechanochemical feedback and crowding. bioRxiv.

[bib32] Ohoka A (2015). EPLIN is a crucial regulator for extrusion of RasV12-transformed cells. Journal of Cell Science.

[bib33] Park JA, Kim JH, Bi D, Mitchel JA, Qazvini NT, Tantisira K, Park CY, McGill M, Kim SH, Gweon B, Notbohm J, Steward R, Burger S, Randell SH, Kho AT, Tambe DT, Hardin C, Shore SA, Israel E, Weitz DA, Tschumperlin DJ, Henske EP, Weiss ST, Manning ML, Butler JP, Drazen JM, Fredberg JJ (2015). Unjamming and cell shape in the asthmatic airway epithelium. Nature Materials.

[bib34] Pérez-Verdugo F, Maniou E, Galea GL, Banerjee S (2026). Mechanosensitive feedback organizes cell shape and motion during hindbrain neuropore morphogenesis. Current Biology.

[bib35] Pothapragada SP (2022). Matrix mechanics regulates epithelial defence against cancer by tuning dynamic localization of filamin. Nature Communications.

[bib36] Rozman J, Yeomans JM, Sknepnek R (2023). Shape-tension coupling produces nematic order in an epithelium vertex model. Physical Review Letters.

[bib37] Sadati M, Qazvini NT, Krishnan R, Park CY, Fredberg JJ (2013). Collective migration and cell jamming. Differentiation; Research in Biological Diversity.

[bib38] Salmon H, Remark R, Gnjatic S, Merad M (2019). Host tissue determinants of tumour immunity. Nature Reviews. Cancer.

[bib39] Sasaki A (2018). Obesity suppresses cell-competition-mediated apical elimination of RasV12-transformed cells from epithelial tissues. Cell Reports.

[bib40] Schindelin J, Arganda-Carreras I, Frise E, Kaynig V, Longair M, Pietzsch T, Preibisch S, Rueden C, Saalfeld S, Schmid B, Tinevez JY, White DJ, Hartenstein V, Eliceiri K, Tomancak P, Cardona A (2012). Fiji: an open-source platform for biological-image analysis. Nature Methods.

[bib41] Schneider G, Schmidt-Supprian M, Rad R, Saur D (2017). Tissue-specific tumorigenesis: context matters. Nature Reviews. Cancer.

[bib42] Sinjab A, Han G, Wang L, Kadara H (2020). Field carcinogenesis in cancer evolution: what the cell is going on. Cancer Research.

[bib43] Skamrahl M, Schünemann J, Mukenhirn M, Pang H, Gottwald J, Jipp M, Ferle M, Rübeling A, Oswald TA, Honigmann A, Janshoff A (2023). Cellular segregation in cocultures is driven by differential adhesion and contractility on distinct timescales. PNAS.

[bib44] Stringer C, Wang T, Michaelos M, Pachitariu M (2021). Cellpose: a generalist algorithm for cellular segmentation. Nature Methods.

[bib45] Sussman DM, Schwarz JM, Marchetti MC, Manning ML (2018). Soft yet sharp interfaces in a vertex model of confluent tissue. Physical Review Letters.

[bib46] Thielicke W, Sonntag R (2021). Particle image velocimetry for MATLAB: Accuracy and enhanced algorithms in PIVlab. Journal of Open Research Software.

[bib47] Vogelstein B, Papadopoulos N, Velculescu VE, Zhou S, Diaz LA, Kinzler KW (2013). Cancer genome landscapes. Science.

[bib48] Yamashita S (2018). *Genetic* and epigenetic alterations in normal tissues have differential impacts on cancer risk among tissues. PNAS.

[bib49] Yang X, Bi D, Czajkowski M, Merkel M, Manning ML, Marchetti MC (2017). Correlating cell shape and cellular stress in motile confluent tissues. PNAS.

[bib50] Zack TI, Schumacher SE, Carter SL, Cherniack AD, Saksena G, Tabak B, Lawrence MS, Zhsng CZ, Wala J, Mermel CH, Sougnez C, Gabriel SB, Hernandez B, Shen H, Laird PW, Getz G, Meyerson M, Beroukhim R (2013). Pan-cancer patterns of somatic copy number alteration. Nature Genetics.

