# Supplementary material for: Heterotypic interfacial tension between oncogenic and wild-type populations forms the mechanical basis of tissue-specific oncogenesis in epithelia
Source: eLife. 2026 Aug 6;14:RP106893. doi: 10.7554/eLife.106893 (PMC13446903; doi:10.7554/eLife.106893)
Supplement: Peer review history

# Editor report: eLife Assessment

**DOI:** 10.7554/eLife.106893.4.sa0

**Authors:** Pierre Sens

**Affiliations:** Institut Curie, CNRS UMR168 France

**Keywords:** Convincing, Important

This **important** study reports that an oncogenic population in an epithelium can either be repressed or spread, depending on the tissues. This work provides **convincing** evidence, supported by pharmacological perturbations and numerical simulations using the vertex model, that the principle of "high heterotypic interfacial tension" that appears to drive cell sorting and tissue segregation in embryonic models similarly applies to cancer cell behaviour.

# Referee report: Reviewer #1 (Public review)

**DOI:** 10.7554/eLife.106893.4.sa1

[Editors' note: this version has been assessed by the Reviewing Editor without further input from the original reviewers. The authors have addressed the comments raised in the previous round of review.]

Summary:

The behaviour of cells expressing constitutively active HRas is examined in mosaic monolayers, both in MCF10a breast epithelial and Beas2b bronchial epithelial cell lines, mimicking the potential initial phase of development of carcinoma. Single HRas-positive cells are excluded from MCF10a but not Beas2b monolayers. Most interestingly, however, when in groups, these cells are not excluded, but rather sharply segregated within a MCF10a monolayer. In contrast, they freely mix with wt Beas2b cells. Biophysical analysis identifies high tension at heterotypic interfaces between HRas and wild-type cells as the likely reason for segregation of MCF10a cells. The hypothesis is supported experimentally, as myosin inhibition abolishes segregation. The probable reason for lack of segregation in the bronchial epithelium is to be found in the different intrinsic properties of these cells, which form a looser tissue with lower basal actomyosin activity. The behaviour of single cells and groups is recapitulated in a vortex model based on the principle of differential interfacial tension, under the condition of high heterotypic interfacial tension.

Strengths:

Despite being long recognized as a crucial event during cancer development, segregation of oncogenic cells has been a largely understudied question. This nice work addresses the mechanics of this phenomenon through a straightforward experimental design, applying the biophysical analytical approaches established in the field of morphogenesis. Comparison between two cell types provides some preliminary clues on the diversity of effects in various cancers.

# Referee report: Reviewer #2 (Public review)

**DOI:** 10.7554/eLife.106893.4.sa2

Summary:

The authors investigate the behavior of oncogenic cells in mammary and bronchial epithelia. They observe that individual oncogenic cells are preferentially excluded from the mammary epithelium, but they remain integrated in the bronchial epithelium. They also observe that clusters of oncogenic cells form a compact cluster in mammary epithelium, but they disperse in the bronchial epithelium. The authors demonstrate experimentally and in the vertex model simulations that the difference in observed behavior is due to the differential tension between the mutant and wild-type cells due to a differential expression of actin and myosin.

Strengths:

(1) Very detailed analysis of experiments to systematically characterize and quantify differences between mammary and bronchial epithelia.

(2) Detailed comparison between the experiments and vertex model simulations to identify the differential cell line tension between the oncogenic and wild-type cells as one of the key parameters that are responsible for the different behavior of oncogenic cells in mammary and bronchial epithelia.

# Author comment: Author response

**DOI:** 10.7554/eLife.106893.4.sa3

**Authors:** Amrapali Datta, Phanindra Dewan, Aswin Anto Puthoor, Tanya Chhabra, Tanishq Tejaswi, Sindhu Muthukrishnan, Akshar Rao, Sumantra Sarkar, Medhavi Vishwakarma

**Affiliations:** Indian Institute of Science Bangalore Bangalore India; Indian Institute of Science Bangalore Bangalore India; Indian Institute of Science Bangalore Bangalore India; Syracuse University Syracuse United States; Indian Institute of Science Bangalore Bangalore India; Indian Institute of Science Bangalore Bangalore India; Indian Institute of Science Bangalore Bangalore India; Indian Institute of Science, Bangalore Bengaluru India; Indian Institute of Science Bangalore Bangalore India

The following is the authors’ response to the previous reviews

**Public Reviews:**

**Reviewer #1 (Public review):**

Summary:

The behaviour of cells expressing constitutively active HRas is examined in mosaic monolayers, both in MCF10a breast epithelial and Beas2b bronchial epithelial cell lines, mimicking the potential initial phase of development of carcinoma. Single HRas-positive cells are excluded from MCF10a but not Beas2b monolayers. Most interestingly, however, when in groups, these cells are not excluded, but rather sharply segregated within a MCF10a monolayer. In contrast, they freely mix with wt Beas2b cells. Biophysical analysis identifies high tension at heterotypic interfaces between HRas and wild-type cells as the likely reason for segregation of MCF10a cells. The hypothesis is supported experimentally, as myosin inhibition abolishes segregation. The probable reason for lack of segregation in the bronchial epithelium is to be found in the different intrinsic properties of these cells, which form a looser tissue with lower basal actomyosin activity. The behaviour of single cells and groups is recapitulated in a vortex model based on the principle of differential interfacial tension, under the condition of high heterotypic interfacial tension.

Strengths:

Despite being long recognized as a crucial event during cancer development, segregation of oncogenic cells has been a largely understudied question. This nice work addresses the mechanics of this phenomenon through a straightforward experimental design, applying the biophysical analytical approaches established in the field of morphogenesis. Comparison between two cell types provides some preliminary clues on the diversity of effects in various cancers.

Weaknesses:

Although not calling into question the main message of this study, there are a few issues that one may want to address:

(1) One may be careful in interpreting the comparison between MCF10a and Beas2b cells as used in this study. The conditions may not necessarily be representative of the actual properties of breast and bronchial epithelia. How much of the epithelial organization is reconstituted under these experimental conditions remains to be established. This is particularly obvious for bronchial cells, which would need quite specific culture conditions to build a proper bronchial layer. In this study, they seemed to be on the verge of a mesenchymal phenotype (large gaps, huge protrusions, cells growing on top of each other, as mentioned in the manuscript).

As an alternative to Beas2b, comparison of MCF10a with another cell line capable of more robust in vitro epithelial organization, but ideally with different adhesive and/or tensile properties, would be highly interesting, as it may narrow down the parameters involved in segregation of oncogenic cells.

(2) While the seminal description of tissue properties based on interfacial tensions (Brodland 2002) is clearly key to interpreting these data, the actual "Differential Interfacial Tension Hypothesis" poses that segregation results from global differences, i.e., juxtaposition of two tissues displaying different intrinsic tensions. On the contrary, the results of the present work support a different scenario, where what counts is the actual difference in tension ALONG the tissue boundary, in other words, that segregation is driven by high HETEROTYPIC interfacial tension. This is an important distinction that should be clarified.

(3) Related: The fact that actomyosin accumulates at the heterotypic interface is key here. It would be quite informative to better document the pattern of this accumulation, which is not clear enough from the images of the current manuscript: Are we talking about the actual interface between mutant and wt cells (membrane/cortex of heterotypic contacts)? Or is it more globally overactivated in the whole cell layer along the border? Some better images and some quantification would help.

(4) In the case of Beas2b cells, mutant cells show higher actin than wt cells, while actin is, on the contrary, lower in mutant MCF10a cells (Figure 2b). Has this been taken into account in the model? It may be in line with the idea that HRas may have a different action on the two cell types, a possibility that would certainly be worth considering and discussing.

Comments on revisions:

There is still one last point that should be made even clearer:

The system is being modelled based on the principle of INTERFACIAL TENSION, a description pioneered by the works of Steinberg and of Harris, and nicely conceptualized by Brodland (2002). Now the observed behaviour is a perfect case of sorting based on higher interfacial tension AT the boundary between cell types (with nice additional documentation of local actin and myosin enrichment in the revised manuscript). What needs to be made crystal clear it that this is NOT equivalent to the model of DITH ("DIFFERENTIAL INTERFACIAL TENSION HYPOTHESIS)" (Brodland 2002, Krieg et al 2008). It is important to stop using DITH in this context, as it leads to confusion and misinterpretations. Indeed, DITH predicts cell/tissue sorting based on differences in interfacial tension WITHIN the two cell types. While DITH accounts for relative POSITIONING (one tissue engulfing the other), it is now established that this is not the motor for cell sorting and tissue segregation, the key parameter is being heterotypic tension at the heterotypic interface. I thus invite the authors to avoid the terms "differential"/DITH, and rather use either "interfacial tension", or specifically to "HIGH HETEROTYPIC INTERFACIAL TENSION".

Related: the authors correctly cite Canty et al NatComm2017 when discussing this phenomenon. I suggest to add an additional key supporting reference "D.M. Sussman, J.M. Schwarz, M.C. Marchetti, M.L. Manning, Soft yet sharp interfaces in a vertex model of confluent tissue, Phys. Rev. Letters 120 (2018) 058001". One may also include another pioneer work in Drosophila is "M. Aliee, J.C. Roper, K.P. Landsberg, C. Pentzold, T.J. Widmann, F. Julicher, C. Dahmann, Physical mechanisms shaping the Drosophila dorsoventral compartment boundary, Curr. Biol. 22 (2012) 967-976."

We thank the reviewer for this important clarification. We fully agree that the mechanism underlying the observed segregation in our system is best described in terms of elevated heterotypic interfacial tension, rather than the classical Differential Interfacial Tension Hypothesis (DITH). As the reviewer correctly points out, DITH in its original formulation refers to differences in intrinsic interfacial tensions within each cell population, which primarily governs relative positioning (e.g., tissue engulfment), rather than the local sorting dynamics we observe here.

In contrast, our experimental and modeling results support a scenario in which segregation is driven by increased tension specifically at heterotypic interfaces between HRasV12 and wild-type cells. We agree that continued use of the term “Differential interfacial tension” in this context may lead to conceptual ambiguity.

Accordingly, we have revised the manuscript throughout to replace references to “differential interfacial tension” with more precise terminology, namely “interfacial tension” or “heterotypic interfacial tension”, wherever appropriate. We have also updated the Discussion to explicitly clarify this distinction and its implications for interpreting our results.

We thank the reviewer for suggesting additional relevant literature which have now included.

**Reviewer #2 (Public review):**

Summary:

The authors investigate the behavior of oncogenic cells in mammary and bronchial epithelia. They observe that individual oncogenic cells are preferentially excluded from the mammary epithelium, but they remain integrated in the bronchial epithelium. They also observe that clusters of oncogenic cells form a compact cluster in mammary epithelium, but they disperse in the bronchial epithelium. The authors demonstrate experimentally and in the vertex model simulations that the difference in observed behavior is due to the differential tension between the mutant and wild-type cells due to a differential expression of actin and myosin.

Strengths:

Very detailed analysis of experiments to systematically characterize and quantify differences between mammary and bronchial epithelia

Detailed comparison between the experiments and vertex model simulations to identify the differential cell line tension between the oncogenic and wild-type cells as one of the key parameters that are responsible for the different behavior of oncogenic cells in mammary and bronchial epithelia

Weaknesses:

It is unclear what is the mechanistic origin of the shape-tension coupling, which is used in the vertex model, and how important that coupling is for the presented results. Authors claim that the shape-tension coupling is due to the anisotropic distribution of stress fibers when cells are under external stress. It is unclear why the stress fibers should affect an effective line tension on the cell boundaries and why the stress fibers should be sensitive to the magnitude of the internal isotropic cell pressure. In experiments, it makes sense that stress fibers form when cells are stretched. Similar stress fibers form when cytoskeleton or polymer networks are stretched. It is unclear why the stress fibers should be sensitive to the magnitude of internal isotropic cell pressure. If all the surrounding cells have the same internal pressure, then the cell would not be significantly deformed due to that pressure and stress fibers would not form. Authors should better justify the use of the shape-tension coupling in the model, since most of the observed behavior is already captured by the differential tension even if there is no shape-tension coupling.

We thank the reviewer for this comment. We agree that we did not provide a mechanistic origin for the shape-tension coupling. In our model, stress fiber formation, along with actin ring formation, indicated that cells at the interface were elongated. Hence, we hypothesised that an interfacial force could induce nematic alignment at the interface. However, such an activity would only be feasible if the interface interaction were sufficiently high. Thus, the isotropic pressure at the heterotypic interface served as a proxy for cell-cell interactions in our model. However, inspired by recent work [1], we have tested whether activation of cells at the interface by shear stress would produce similar results. Exploring this aspect will require additional simulations.

(1) Pérez-Verdugo, F., Maniou, E., Galea, G. L., & Banerjee, S. (2026). Mechanosensitive feedback organizes cell shape and motion during hindbrain neuropore morphogenesis. Current Biology.

The observed difference of shape indices between the interfacial and bulk cells in simulations in the absence of differential line tension is concerning. This suggests that either there are not enough statistics from the simulations or that something is wrong with the simulations. For all presented simulation results, the authors should repeat multiple simulations and then present both averages and standard deviations. This way it would be easier to determine whether the observed differences in simulations are statistically significant.

The observed differences in shape indices between interfacial and bulk cells in simulations in the zero-line-tension case (Lambda=0) remain non-zero at the zero-stress threshold because the interface cells are still subject to the shape-dependent contribution gamma_ij, since the current model treats gamma_ij as independent of Lambda. We are exploring the possible relationship between Lambda and gamma_ij, and we will update this in the next version of the manuscript.

**Recommendations for the authors:**

The editor recommends considering the new comment made by reviewer #1 in his/her report:

"There is still one last point that should be made even more clear:

The system is being modelled based on the principle of INTERFACIAL TENSION, a description pioneered by the works of Steinberg and of Harris, and nicely conceptualized by Brodland (2002). Now the observed behaviour is a perfect case of sorting based on higher interfacial tension AT the boundary between cell types (with nice additional documentation of local actin and myosin enrichment in the revised manuscript). What needs to be made crystal clear it that this is NOT equivalent to the model of DITH ("DIFFERENTIAL INTERFACIAL TENSION HYPOTHESIS)" (Brodland 2002, Krieg et al 2008). It is important to stop using DITH in this context, as it leads to confusion and misinterpretations. Indeed, DITH predicts cell/tissue sorting based on differences in interfacial tension WITHIN the two cell types. While DITH accounts for relative POSITIONING (one tissue engulfing the other), it is now established that this is not the motor for cell sorting and tissue segregation, the key parameter is being heterotypic tension at the heterotypic interface. I thus invite the authors to avoid the terms "differential"/DITH, and rather use either "interfacial tension", or specifically to "HIGH HETEROTYPIC INTERFACIAL TENSION".

Related: the authors correctly cite Canty et al NatComm2017 when discussing this phenomenon. I suggest to add an additional key supporting reference "D.M. Sussman, J.M. Schwarz, M.C. Marchetti, M.L. Manning, Soft yet sharp interfaces in a vertex model of confluent tissue, Phys. Rev. Letters 120 (2018) 058001". One may also include another pioneer work in Drosophila is "M. Aliee, J.C. Roper, K.P. Landsberg, C. Pentzold, T.J. Widmann, F. Julicher, C. Dahmann, Physical mechanisms shaping the Drosophila dorsoventral compartment boundary, Curr. Biol. 22 (2012) 967-976."

Please see response to Reviewer 1

**Reviewer #2 (Recommendations for the authors):**

The authors have improved the manuscript and addressed some of my concerns. However, some of the questions were not adequately addressed.

(1) I appreciate additional justification regarding the need for the shape-tension coupling in the vertex model. However, the authors have not answered my question regarding why the shape-tension coupling model should be sensitive to the magnitude of the internal isotropic cell pressure. In experiments, it makes sense that stress fibers form when cells are stretched, but it is unclear why the stress fibers should be sensitive to the magnitude of internal isotropic cell pressure. If all the surrounding cells have the same internal pressure, then the cell would not be significantly deformed due to that pressure, and stress fibers would not form.

We thank the reviewer for pointing this out. We agree that we did not provide a mechanistic origin for the shape-tension coupling. In our model, stress fiber formation, along with actin ring formation, indicated that cells at the interface were elongated. Hence, we hypothesized that an interfacial force could induce nematic alignment at the interface. However, such an activity would only be feasible if the interface interaction were sufficiently high. Thus, the isotropic pressure at the heterotypic interface served as a proxy for cell-cell interactions in our model.

However, inspired by recent work [1], we have tested whether activation of cells at the interface by shear stress would produce similar results. Exploring this aspect will require additional simulations.

(1) Pérez-Verdugo, F., Maniou, E., Galea, G. L., & Banerjee, S. (2026). Mechanosensitive feedback organizes cell shape and motion during hindbrain neuropore morphogenesis. Current Biology.

(2) I appreciate that the authors provided additional statistics related to simulations. I am still very concerned about the observed difference in the shape indices between the cells at the interface and the bulk, when the interfacial line tension is exactly zero (Lambda=0). In that case, the cells at the interface and at the boundary are identical, and there should be no difference in the shape indices. Are cells at the interface for the zero-line tension case (Lambda=0) still subject to the shape dependent contribution gamma_ij? If that contribution is still included for the cells at the interface, then this could explain why cells at the interface are still different from cells in the bulk even when Lambda=0.

The observed differences in shape indices between interfacial and bulk cells in simulations in the zero-line-tension case (Lambda=0) remain non-zero at the zero-stress threshold because the interface cells are still subject to the shape-dependent contribution gamma_ij, since the current model treats gamma_ij as independent of Lambda. We are exploring the possible relationship between Lambda and gamma_ij, and we will update this in the next version of the manuscript.

(3) Authors included several additional supplemental figures (Figs. S4, S5, S6, S7) , but they are not discussed in the manuscript text. These new supplemental figures were only discussed in the rebuttal letter. These figures should also be discussed in the manuscript text.

We have cited the new supplementary figures in the main text.

(4) Authors have answered in the rebuttal letter what experimental data was used in Fig. 4c. This information also needs to be provided in the manuscript text.

We have added this information in the caption of Figure 4

(5) Supplementary Figure 3 is missing. That figure got moved to the appendix.

This has been rectified in the Supplementary file and the citations have been updated accordingly in the main text.

(6) At the end of section 4 in the main text, the authors introduced a new sentence regarding simulations of the vertex model with interfacial tension and mechanochemical feedback. The details of that model are described in the appendix, but it would be helpful to add a sentence or two already in the main text describing what is the mechanism of the mechanochemcial feedback.

We have added a line describing the mechanism of mechanochemical feedback.

(7) In the definition of the eccentricity, 'a' should be the minor axis and 'b' the major axis, i.e., 'a' and 'b' should be swapped.

We have corrected this.

(8) There is a typo at the end of the vertex model description in the methods section. "The details of the shape-tension coupling is described in the interface." The word interface should be an appendix.

We have fixed the typo.

(9) In the appendix section describing the shape-tension coupling, the authors should explain how the cell's director n is defined.

We have added a line in the appendix section describing shape-tension coupling explaining how the cell’s director n is defined.

(10) In Appendix Fig. 1, the two angles are defined as theta and theta' but the figure caption is defining angles theta_1 and theta_2. These angles need to be consistent.

This has been fixed.
